# A Distinct, Flocculent, Acidogenic Microbial Community Accompanies Methanogenic Granules in Anaerobic Digesters

**DOI:** 10.1128/Spectrum.00784-21

**Published:** 2021-11-10

**Authors:** Simon Mills, Anna Christine Trego, Piet N. L. Lens, Umer Zeeshan Ijaz, Gavin Collins

**Affiliations:** a Microbial Communities Laboratory, School of Natural Sciences, National University of Ireland Galway, Galway, Ireland; b IETSBIO3 Laboratory, National University of Ireland, Galway, Galway, Ireland; c Infrastructure and Environment, School of Engineering, The University of Glasgow, Glasgow, United Kingdom; d Ryan Institute, National University of Ireland Galway, Galway, Ireland; Temasek Life Sciences Laboratory

**Keywords:** anaerobic digestion, biofilm, community succession, methanogenic granules, anaerobic granules, 16S rRNA, acidogenic

## Abstract

The formation of dense, well-settling methanogenic granules is essential for the operation of high-rate, up-flow anaerobic bioreactors used for wastewater treatment. Granule formation (granulation) mechanisms have been previously proposed, but an ecological understanding of granule formation is still lacking. Additionally, much of the current research on granulation only examines the start-up phase of bioreactor operation, rather than monitoring the fate of established granules and how new granules emerge over time. This paper, therefore, attempts to provide an insight into the microbial ecology of granule formation outside the start-up phase of bioreactor operation and develop an ecological granulation model. The microbial communities of granules actively undergoing growth, breakage, and reformation were examined, and an ecological granulation model was proposed. A distinct pregranular microbial community, with a high proportion of acidogenic organisms, such as the *Streptococcaceae*, was identified and suggested to have a role in initiating granulation by providing simpler substrates for the methanogenic and syntrophic communities which developed during granule growth. After initial granule formation, deterministic influences on microbial community assembly increased with granule size and indicated that microbial community succession was influenced by granule growth, leading to the formation of a stepwise ecological model for granulation.

**IMPORTANCE** Complex microbial communities in engineered environments can aggregate to form surface-attached biofilms. Others form suspended biofilms, such as methanogenic granules. The formation of dense, methanogenic granules underpins the performance of high-rate, anaerobic bioreactors in industrial wastewater treatment. Granule formation (granulation) has been well studied from a physico-chemical perspective, but the ecological basis is poorly understood. We identified a distinct, flocculent, microbial community, which was present alongside granules, comprising primary consumers likely key in providing simpler substrates to granules. This flocculent community is understudied in anaerobic digestion and may initiate, or perpetuate, granule formation. We propose that it may be possible to influence bioreactor performance (e.g., to regulate volatile fatty acid concentrations) by manipulating this community. The patterns of microbial community diversity and assembly revealed by the study indicate that cycles of granule growth and breakage lead to overall diversification of the bioreactor meta-community, with implications for bioreactor process stability.

## INTRODUCTION

Methanogenic consortia underpin efficient waste-to-energy conversion in up-flow anaerobic bioreactors, in which microbial communities may form anaerobic sludge granules. Such granules are spherical microbial aggregates approximately 0.5 to 4 mm in size, each containing all of the necessary organisms for the complete conversion of organic contaminants to methane (1). Retention of well-functioning microbial biomass is essential for such bioreactors, and the immobilization of microbial communities into dense, settleable granules allows high up-flow velocities to be applied without biomass washout (2). Difficulties in achieving granulation, or even granule disintegration and washout, have been reported to inhibit up-flow anaerobic bioreactor performance (3).

A comprehensive outline of early granulation mechanisms was previously published (4), and research on granulation is still under way (5–7). However, the process of granule formation (granulation), granule disintegration, and regranulation requires further investigation. Indeed, these mechanisms are often without ecological explanations and rarely take into account the fate of established granules. It is unlikely that granules, once formed, remain intact for extended periods, and they probably break apart (8). Consequently, the fate of the resultant granule particles and the release of internal microbial populations—previously isolated from other granules—is likely important for granulation and a driver of microbial diversity in bioreactors.

The formation of traditional, surface-attached biofilms is well defined and widely accepted (9). Even though granules are considered biofilms (9–11), the five main steps of traditional biofilm formation (initial attachment to surfaces, irreversible attachment through exopolysaccharide [EPS] production, formation of early biofilm architecture, biofilm architecture maturation, and dispersal [12]) cannot be applied directly to spherical biofilms formed by auto-immobilization. The importance of EPS production has been established (13, 14), and the concentric layers (15) and channels (16) in granules could be considered analogous to architectural features of traditional biofilms. However, initial formation and dispersal steps clearly differ in granules, due to their suspended nature. To address those unknowns, it is essential to study growing granules of different sizes, i.e., small (young) and large (older). Granules are rarely sampled by size, and it is unknown whether microbial communities change during granule growth and how cycles of growth and breakage influence the ecology of the whole bioreactor community. Studying granules undergoing active growth and disintegration would address this knowledge gap and allow patterns in community succession to be identified, informing an ecological granulation model.

Patterns of community succession have previously been observed in complex microbial communities (17, 18). Microbial community succession on organic marine particles has previously been identified (17), whereby primary consumers initiated colonization and facilitated the succession of secondary consumers. Similar complementary metabolisms occur widely in anaerobic digestion (19), and cooperation and mutualism are considered drivers of diversity in methanogenic communities (20). It is possible that a sequence of community succession could facilitate granule formation, where planktonic microorganisms along with particulate matter, such as pieces of old, disintegrated granules, aggregate to initiate the development of new, small granules, which then grow through stages of community succession.

In the present study, differently sized granules were sampled from 12 laboratory-scale (2-liter) bioreactors, which were described previously (21), to assess community dynamics during the growth, maturation, and break-up of granules. The aims were to (i) monitor granulation as a continual, dynamic process rather than only during start-up, (ii) characterize the microbial communities associated with granules in different stages of development to observe patterns of community succession, and (iii) develop an ecological granulation model. It was hypothesized that distinct communities would be associated with differently sized granules and different taxa would have specific roles in a community succession granulation model.

## RESULTS

### Influence of size separation on resultant microbial communities.

**FIG 1:**
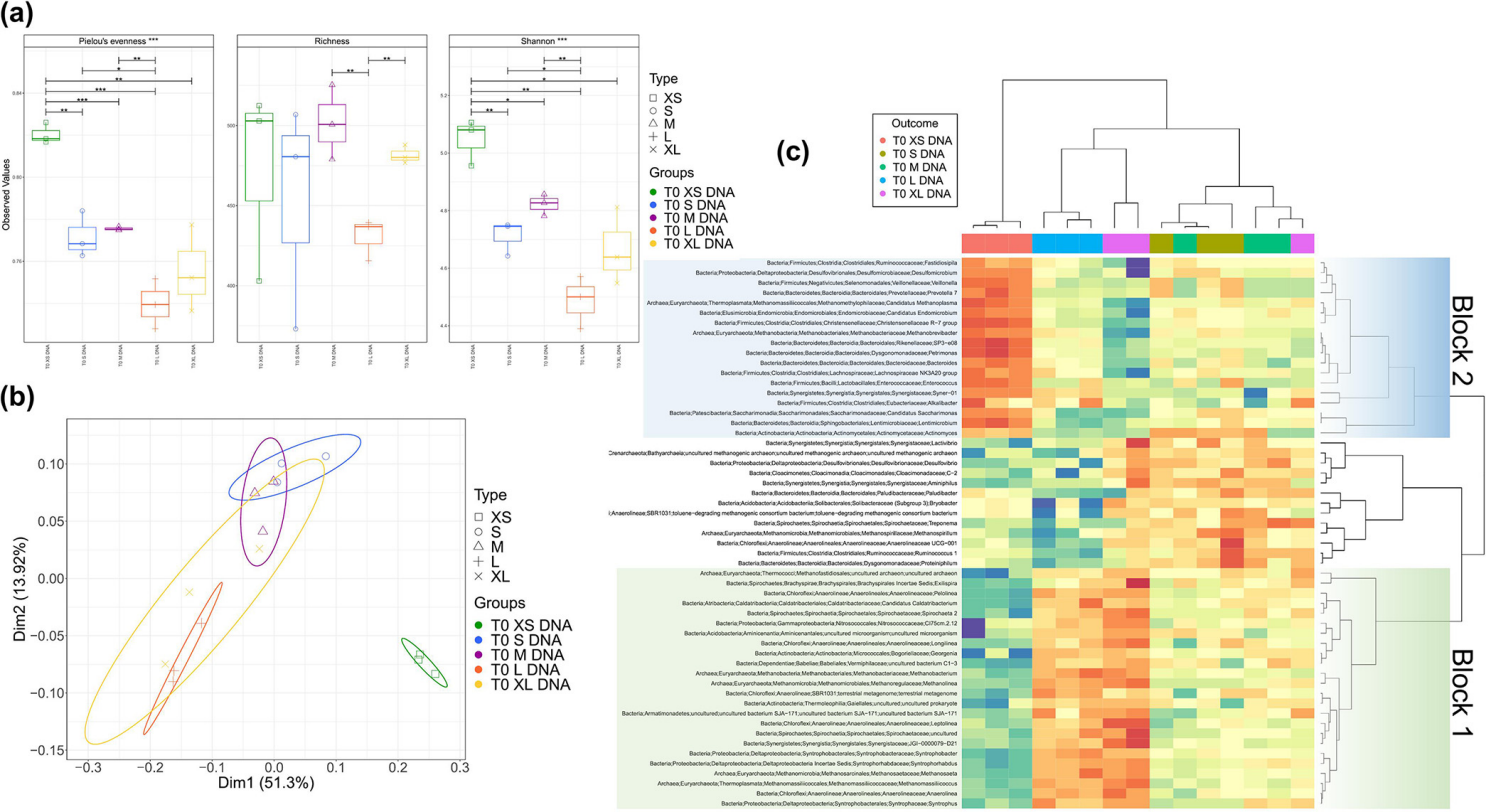
Microbial community analysis of seed sludge. (a) Alpha diversity indices; Pielou’s evenness, rarefied richness and Shannon entropy (lines of significance depict significant differences as follows: *, *P* < 0.05; **, *P* < 0.01; ***, *P* < 0.001 based on ANOVA. (b) Principal-component analysis (Bray-Curtis) of biomass of seed samples grouped by size, where ellipses were drawn using 95% confidence intervals based on standard deviation. (c) Results from the heatmap are based on sPLS-DA of amplicon sequencing data depicting discriminant genera. Rows and columns are ordered according to hierarchical (average linkage) clustering to identify clusters of genera among groups. Block 1 contains discriminant taxa more abundant in L and XL granules, and block 2 contains taxa more abundant in XS biomass.

The microbial communities of the starting (T0)-size bins were analyzed to determine the potential impact of size separation on the evolution of subsequent microbial communities in each reactor. Microbial community evenness and diversity decreased with increasing granule size. Large (L) seed sludge (T0 L DNA) was significantly lower in evenness and diversity than small (S) seed sludge (T0 S DNA), but there was no significant difference in richness (Fig. 1). L and S seed sludge also clustered separately based on principal-coordinate analysis using the Bray-Curtis distance metric (Fig. 1). Discriminant taxa, which can be thought of as a subset of the community which explains most of the variation between samples, were identified by sparse projection to latent structure-discriminant analysis (sPLS-DA). Several of these were more abundant in the large granules than in the small granules, including many methanogenic and syntrophic organisms (Fig. 1). The extrasmall (XS) size fraction clustered separately from all other sizes in sPLS-DA and had higher abundances of genera within the *Firmicutes* and *Bacteroidetes* (Fig. 1).

Once seeded, beta diversity in all reactors evolved in a similar fashion from takedown 1 to takedown 4, regardless of the seed source (see Fig. S1 in the supplemental material). Permutational analysis of variance (PERMANOVA) suggested that seed size explained the least variation in the final microbial communities in comparison to other explanatory variables (reactor, granule size, and takedown), indicating that the effect of initial size separation on the resultant microbial communities was minor in comparison to other factors (Fig. S1).

### General trends in diversity.

**FIG 2:**
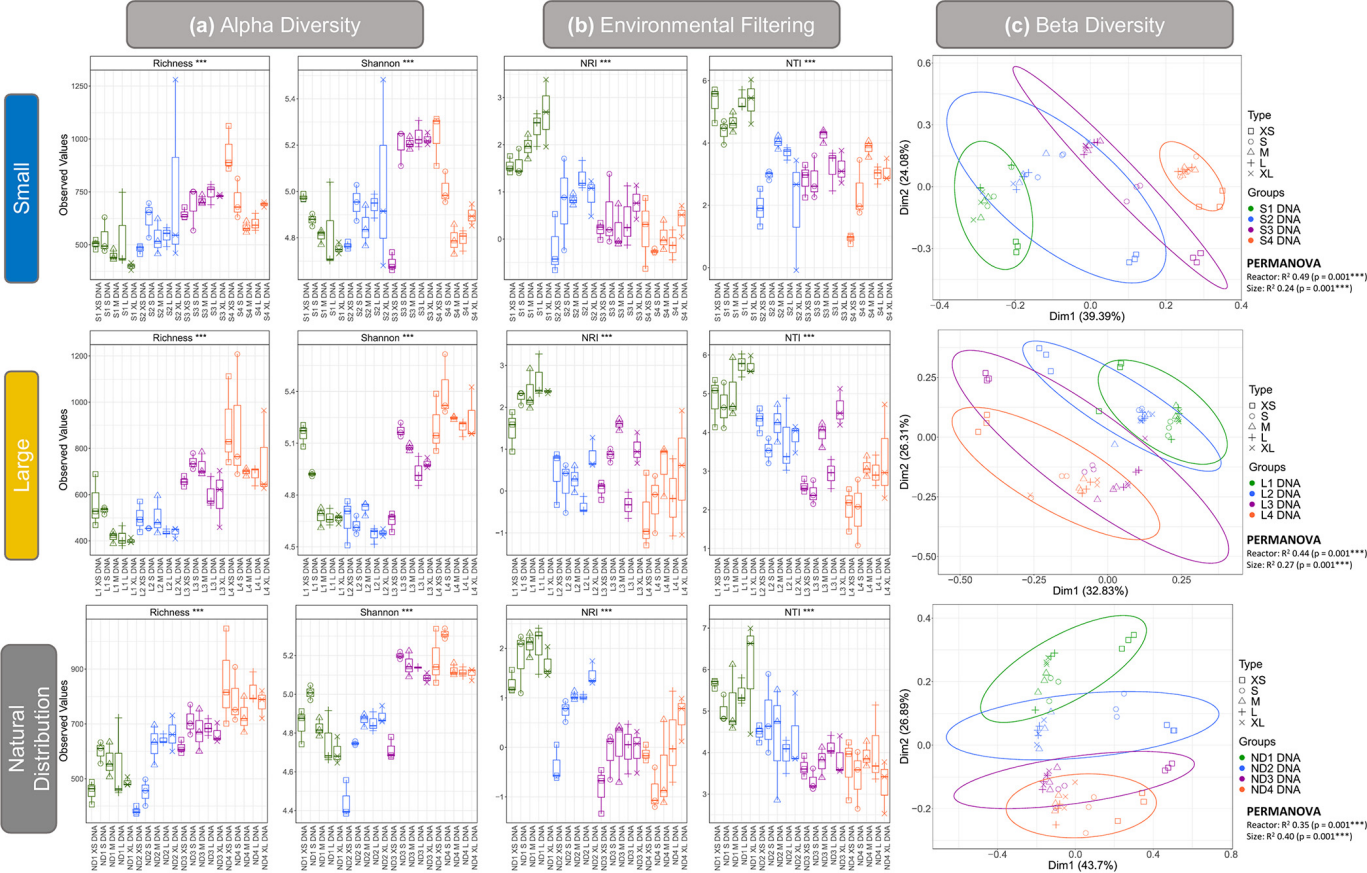
(a) Alpha diversity indices; rarefied richness and Shannon Diversity for all biomass sizes recovered from S, L, and ND reactors. (b) Environmental filtering analysis; NRI and NTI values for all biomass sizes recovered from S, L, and ND reactors. (c) Principal-component analysis with samples grouped by reactor and biomass size, where ellipses were drawn using 95% confidence intervals based on standard deviation. *** indicates that significant differences were found between some of the samples but these have not been specifically marked in the plots.

Trends in alpha diversity were seen with each bioreactor takedown across all bioreactor sets (Fig. 2). Generally, species richness and Shannon entropy increased in granules of all sizes with operation time, from takedown 1 to takedown 4, regardless of seed size (Fig. 2). However, diversity and richness were lower in S, medium (M), L, and extralarge (XL) granules from bioreactor RS4 than the equivalent sizes in bioreactor RS3. Beta diversity analysis showed community succession over time and clustering with respect to reactor and granule size (Fig. 2). Levels of environmental pressure were assessed by determining the phylogenetic distances within each sample using the nearest taxa index (NTI) and net relatedness index (NRI). A positive value for both metrics indicates that amplicon sequencing variants (ASVs) within a sample are more closely related than could be expected to occur by chance. This indicates whether the community structure was stochastic (i.e., driven by competition among taxa or ecological drift) or deterministic (i.e., driven by strong external environmental pressure), where higher values indicate more deterministic influences on the community. High levels of environmental pressure (>2 NTI) were observed in all samples, and a general trend of increasing environmental pressure with granule size was apparent (Fig. 2). Environmental pressure also decreased with each takedown, as alpha diversity increased. NTI is a preferred metric over NRI because of the presence of phylogenetic signal across short phylogenetic distances (22), which is typical of microbiome studies. Although we have obtained significant results for NRI as well, the focus should be on NTI in terms of environmental filtering.

### Pregranular biomass.

**FIG 3:**
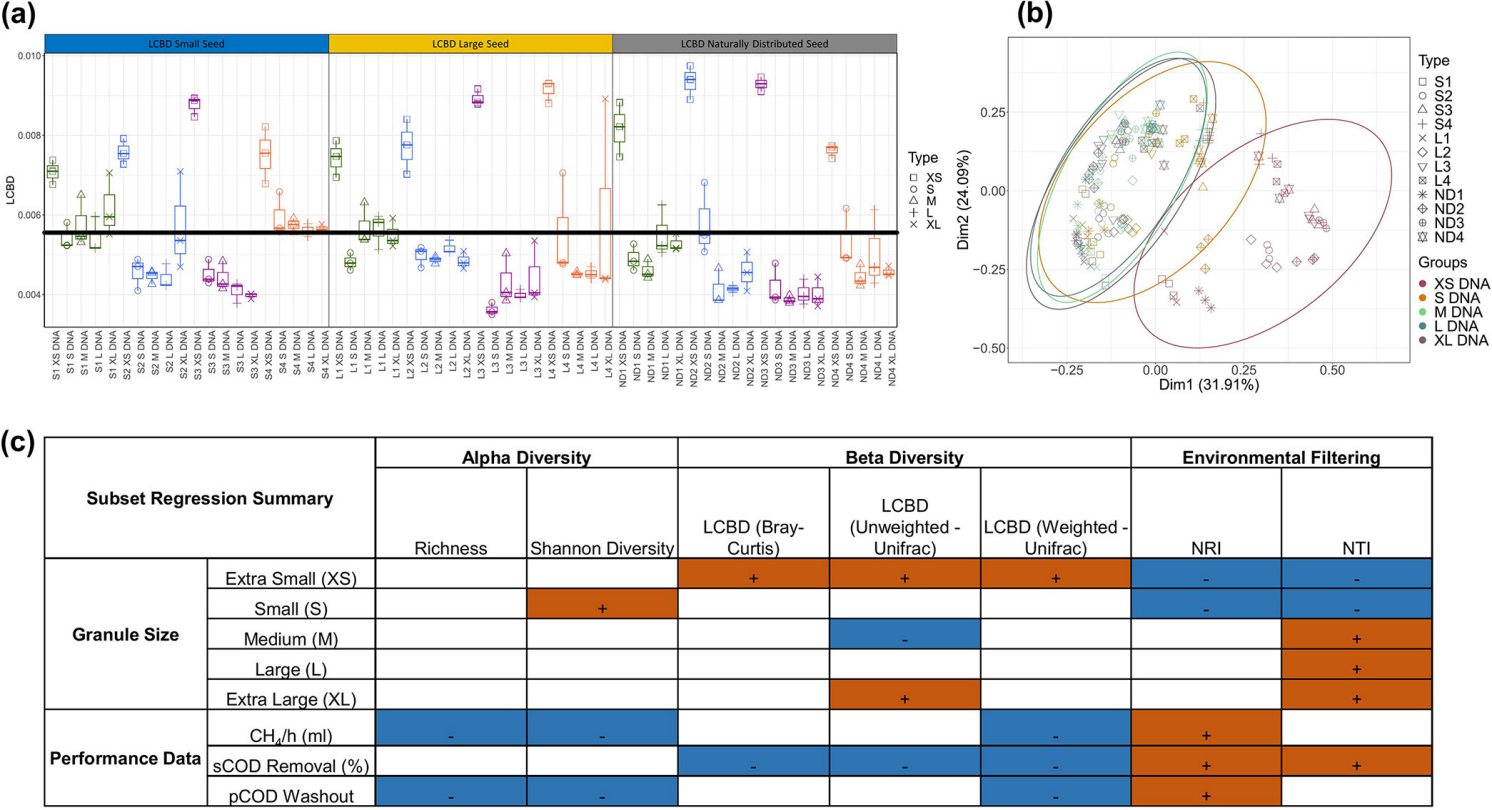
(a) Local contribution to beta diversity (LCBD) of all DNA samples using the Bray-Curtis distance metric. (b) Principle-component analysis (PCA) of all DNA samples, grouped by biomass size, where ellipses were drawn using 95% confidence intervals based on standard deviation. (c) Subset regression analysis assessing the contribution of different sample types to different diversity metrics. Orange (1) boxes indicate a positive contribution by a particular sample type to a particular diversity metric. Blue (–) boxes indicate a negative contribution by a particular sample type to a particular diversity metric. Summary statistics which were used for subset regression analysis are available in Table S1.

XS biomass generally consisted of small particulates and flocculent biomass, which was observed on the top of the sludge bed throughout the trial and was considered to be pregranular. The total community (DNA) of the XS biomass from all reactors was clearly different from the rest of the granule sizes based on principal-component analysis (PCA) (Fig. 3). Of the other sizes, S granules were the most similar to XS, indicating that they may indeed have originated from growth of XS biomass. The contribution of each sample to mean beta diversity was assessed using local contribution to beta diversity (LCBD) analysis, which showed that XS granules contributed the most variation to mean beta diversity (Fig. 3).

The contribution of various extrinsic parameters and different sample classifications to microbiome metrics was assessed using subset regression analysis. It was found that XS biomass made a significant positive contribution to beta diversity and a significant negative contribution to levels of environmental pressure (NRI and NTI) (Fig. 3). This indicates that XS biomass was quite different in composition from other size fractions and less influenced by its environment. S granules also had a negative contribution to overall levels of environmental pressure, whereas M, L, and XL granules generally had a positive contribution.

The community of the granular biomass (S, M, L, and XL) had a higher relative abundance of archaea than the pregranular (XS) biomass (Fig. S2). *Methanosaeta* (the only acetoclastic methanogen in the top 25 taxa), *Methanobacterium*, and *Methanospirillum* spp. were lower in relative abundance in XS biomass than the other granule sizes, whereas *Methanocorpusculum* spp. were more abundant in the XS biomass (Fig. S2). Several bacterial families appeared to be more abundant in the XS biomass, including *Streptococcaceae* and *Eubacteriaceae*, leading to a much higher abundance of *Firmicutes* at the phylum level (Fig. S2). The candidate phylum FCPU426 and candidate genus *Aegiribacteria* (classified at the phylum level in the SILVA132 database) had higher abundances in granular communities, as did *Geobacter* spp.

### Discriminant analysis.

**FIG 4:**
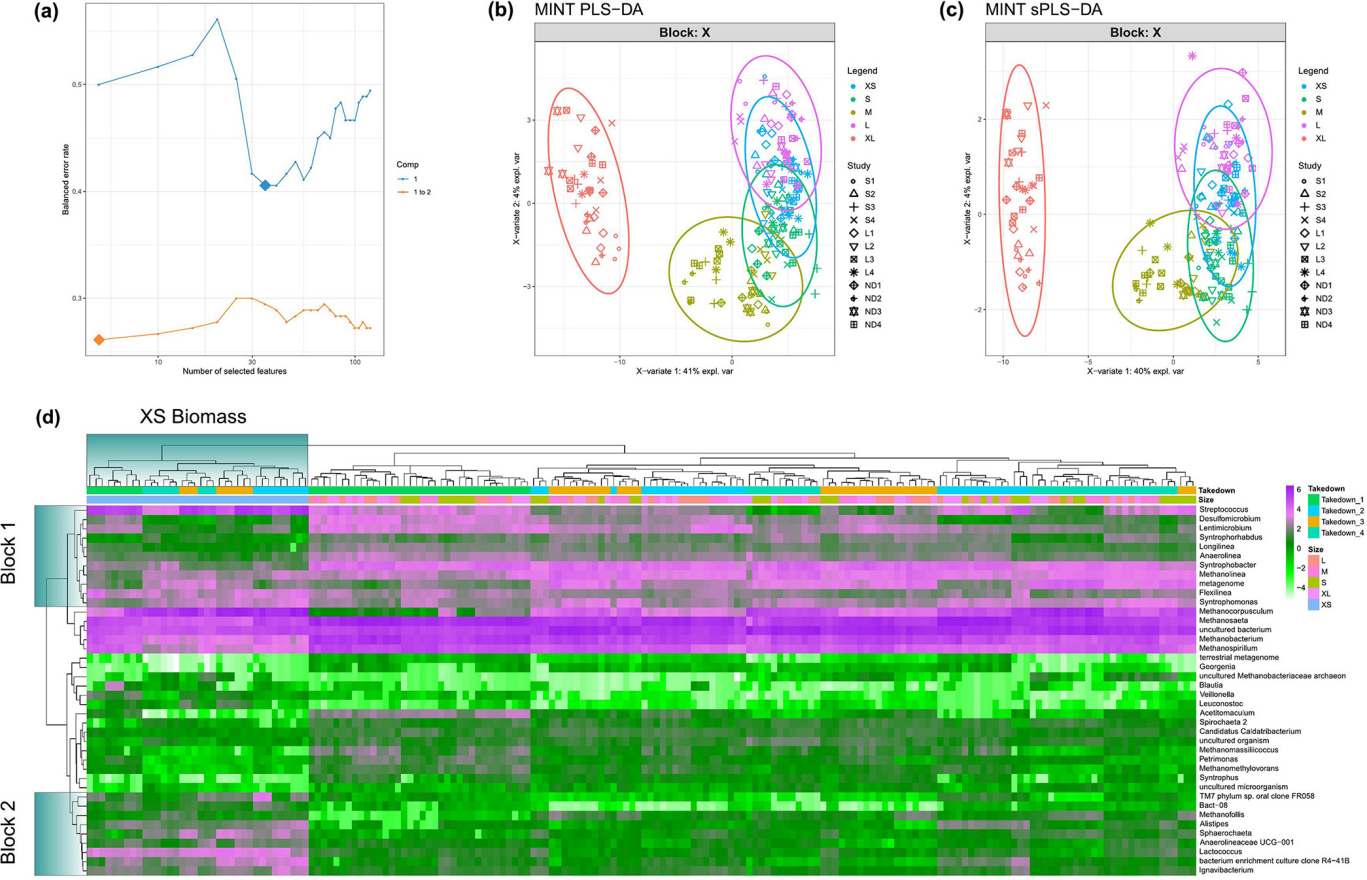
Multivariate integration (MINT) analysis. (a) Classification error rates over the components and the numbers of optimal features (genera) in each component included in the model, chosen by the lowest error rates, which are denoted by diamonds. (b) Ordination of whole ASV table. (c) Ordination of discriminant ASVs only. (d) Heatmap depicting discriminant genera. Rows and columns are ordered according to hierarchical (average linkage) clustering to identify clusters of genera among groups. Blocks 1 and 2 indicate clustering of discriminant genera within the XS biomass group.

A total of 40 discriminant genera which had a major influence in determining beta diversity in the total microbial community (DNA samples only) were identified using multivariate integration (MINT) analyses (Fig. 4). Two blocks (block 1 and block 2) were identified which show clear clustering of genera within the XS biomass. Within block 1, Streptococcus and *Methanocorpusculum* spp. were generally more abundant in XS biomass. Several taxa which were more abundant in XS biomass are also highlighted in block 2, including *Lactococcus*, *Ignavibacterium*, *Spaerochaeta*, and the families *Pedosphaeraceae* and *Rikenellaceae*. Taxa which appeared to be lower in abundance in XS biomass included *Desulfomicrobium*, *Lentimicrobium*, *Longilinea*, *Anaerolinea*, *Syntrophorhabdus*, *Syntrophobacter*, *Methanosaeta*, *Methanobacterium*, *Methanospirillum*, and *Aegiribacteria*.

### Analysis of the active microbial community.

**FIG 5:**
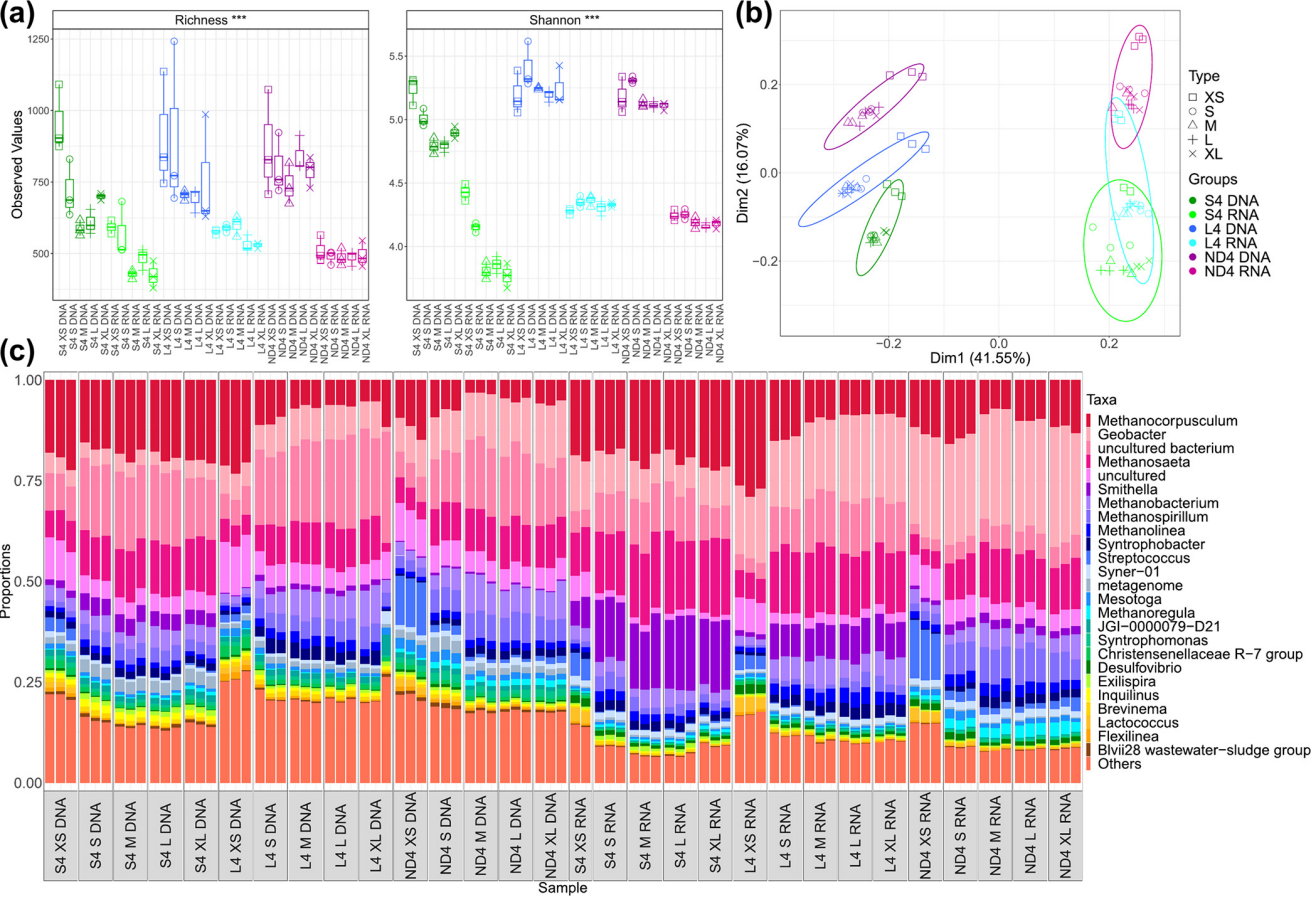
(a) Alpha diversity indices; rarefied richness and Shannon diversity for total (DNA) and active (RNA) communities in RS4, RL4, and RND4. *** indicates that significant differences were found between some of the samples but these have not been specifically marked in the plots. (b) Principal component analysis (PCA) of total (DNA) and active (RNA) communities in RS4, RL4, and RND4. (c) Bar charts depicting the top 25 most abundant genera in the total (DNA) and active (RNA) communities of RS4, RL4, and RND4, where “others” represents taxa outside the top 25 most abundant organisms.

DNA and RNA samples clustered separately in PCA using the Bray-Curtis distance metric (Fig. 5). Similar to analysis of the total community, the active portion of XS biomass clustered separately from the rest of the sizes. Alpha diversity was significantly lower in the active community for all granule sizes in the final takedown.

Of the most abundant taxa, the relative abundance of *Geobacter* increased the most in the active community of all size fractions, as did *Methanosaeta* and *Smithella* sp. A total of 87 discriminant genera were identified by MINT analysis, between the total and active communities of TD4 (Fig. S3). Many of these ASVs were more strongly associated with the active community, indicated in block 1, including the genera *Geobacter*, *Lactococcus*, and *Desulfovibrio*, indicating that these genera may have been more active than is apparent at the DNA level.

### (i) Total versus active communities of each recovered size.

The 87 ASVs identified as a result of total/active community MINT analysis (Fig. S3) were visualized as heat trees to allow for pairwise comparisons of phylogenetic differences between samples (Fig. S4). Multiple members of the Deltaproteobacteria were more abundant in the active community of all granule sizes, including *Geobacter* and *Desulfovibrio* spp. Several other members of the *Proteobacteria*, including *Thauera* and *Methylomonas*, were also generally more relatively abundant in the active community, particularly in S granules. The *Streptococcaceae* family was also universally more abundant in the active community, across all sizes and reactors.

### (ii) Differences in active communities of different sizes.

Direct comparison of the active communities of all granule sizes originating from bioreactors RS4, RL4, and RND4 revealed distinct patterns in the active community (Fig. S5). Active *Streptococcaceae* appear more abundantly in XS biomass than in any other recovered size in any reactor (Fig. S5). *Geobacter* and *Desulfovibrio* were present in similar abundances between sizes. The other *Proteobacteria* mentioned previously, including *Methylomonas* and *Thauera* spp. were more abundant in S granules than in any other granule size (Fig. S5).

## DISCUSSION

### Influence of granule size-separation on resultant microbial communities.

Bioreactors RND1 to 4 were found to generally perform in a more stable manner than RS1 to 4 or RL1 to 4 (21), which could have been due to the presence of the XS biomass in the naturally distributed (ND) seed sludge, which was absent from the size-restricted biomass at day 0. The XS community in the ND seed sludge had higher relative abundances of *Firmicutes* and *Bacteroidetes* (Fig. 1), which contribute to hydrolysis and fermentation in anaerobic digestion (AD) (23). Therefore, alteration of the seed by size separation may have constrained the performance of RL1 to 4 and RS1 to 4 by inhibiting the initial steps of the AD pathway. The XS fraction of the seed was also the most diverse (Fig. 1). Lower diversity in S and L seed sludge could also explain the higher performance variability and lower stability in RS1 to 4 and RL1 to 4 compared to RND1 to 4 (21), as higher diversity is generally thought to promote stability in ecological systems (24). Despite differences in seed sludge diversity and bioreactor performance, beta diversity analysis showed that the final microbial communities clustered by reactor (Fig. 2) and takedown (Fig. S1). This indicates that operation time, rather than seed sludge size distribution, appeared to have caused the most variation in final community composition (Fig. S1). This demonstrates that initial size separation had little effect on the final microbial communities, despite apparently causing some variation in bioreactor performance. This is an important observation in the context of this study, as it means that the microbial communities in the size-restricted biomass behaved similarly to those in the control ND bioreactors despite performance differences, thus, making observations from the size-restricted biomass more applicable to the natural distribution and indicating that the results from RS1 to 4 and RL1 to 4 are not artifacts of the initial size separation.

### Granulation as a driver of diversity.

The development of a full complement of granule sizes (Fig. 1) in RS1 to 4 and RL1 to 4 over time coincided with increased diversity (Fig. 2), possibly leading to more functional redundancy in the microbial community. This poses the question as to what drove the diversification over time and whether it could be controlled to improve bioreactor stability. It is possible that this increase in diversity was simply stochastic. However, all samples had NTI values greater than 2, indicating that deterministic influences played a stronger role in microbial community assembly (25, 26) (Fig. 2). Environmental pressure increased with granule size in all reactors (Fig. 2), and XS and S granules contributed negatively to environmental pressure, whereas M, L, and XL granules contributed positively (Fig. 3). Thus, as granules grew, the respective communities experienced more environmental pressure, perhaps due to factors such as substrate limitation at the granule core (27–29).

It may be the case that cycles of granule growth and breakage influenced net diversity in the bioreactors, in a manner similar to what has been proposed for soil aggregates (30). Soil aggregates were previously proposed to act as evolutionary incubators for microbial communities in which internal microbial communities evolve independently of other aggregates and contribute to net diversity in the bulk soil upon breakage (30). A similar scenario may occur in anaerobic bioreactors where higher levels of environmental pressure in larger granules (Fig. 2 and 3) influence the community during granule growth. We hypothesize that upon breakage, M, L, or XL granules would release independently evolved microbial communities, contributing to net diversification. If this is the case, it might be possible to implement a bioreactor management strategy to promote diversification, and thus stability, by increasing the rate of granule breakage and reformation through mechanical or hydraulic disturbances. However, it is not possible to say for sure based on the current analysis. In addition, stochastic processes such as ecological drift may also play a role in diversification. Indeed, environmental pressure generally decreased over time as alpha diversity increased, indicating that increased stochasticity had a role in diversification despite deterministic influences being dominant. Genome-resolved metagenomics have been applied previously to identify strain-level evolution within aerobic granules (11), and such an approach would be suitable for testing the hypothesis put forward here.

### Distinct pregranular and granular communities.

#### (i) Pregranular community.

It was hypothesized that distinct microbial communities would be associated with different stages of granule growth, informing an ecological community succession granulation model. The microbial community of XS biomass was distinct from those present in the other granule sizes (Fig. 3) and therefore may represent the initial stages of such a model. It is possible that this fraction contributed to initiating granule formation or existed as a background community present throughout the reactors during their operation. At phylum level, *Firmicutes* were more abundant than in the other size fractions (Fig. S2). Firmicutes are widely regarded as an important group in AD, carrying out functions such as acidogenesis (23). *Methanocorpusculum* spp. were the only methanogens within the 25 most abundant taxa more prevalent in the XS than the granular communities (Fig. 4). *Methanocorpusculum* spp. are hydrogenotrophic (31), but little else is known about their role in AD.

The pregranular biomass appeared to be generally present at the top of the sludge bed, due to its very low settling velocity, i.e., <0.02 ms^−1^ (Fig. 1). This community is likely undersampled in lab-scale studies, as granules are generally the biomass of interest. Sampling through reactor ports in the middle of the sludge bed, as is typical, may also lead to underrepresentation of flocculent biomass. However, the planktonic microbiome of a thermophilic upflow anaerobic sludge bed (UASB) was also found to be significantly different from the granular community with a high proportion of *Firmicutes* (32). This community was more susceptible to operational changes than the granular community and was also potentially influenced by the breakup of granules and resultant release of the granule microbial communities (32), in agreement with the results presented here.

The abundance of certain bacteria in XS biomass, such as *Streptococcaceae* (Fig. 4), indicated that the XS biomass may have specialized in acidogenesis. Indeed, acidogenesis was previously suggested to occur on the outermost layer of larger granules or in small granules and flocculent biomass (33–37). Streptococcus spp. have also been suggested to be important for granulation under acidified conditions (8) and at high salinity (7, 38). Their presence in the XS fraction here could indicate that *Streptococcaceae* are more important in initiating granulation than previously assumed, perhaps initiating community succession by providing substrates for acetogenic communities in newly forming granules.

#### (ii) Granular community.

The relative abundance of all methanogens other than *Methanocorpusculum* spp. was higher in the granular community (S to XL granules) than the XS biomass. Notably, *Methanosaeta* spp. were relatively the most abundant organisms in granular communities. Filamentous *Methanosaeta* spp. are known to play an important role in granulation (39).

Several acetogens and methanogens were identified as discriminant taxa, which were more abundant in the granular samples, including *Anaerolinea*, *Longilinea*, *Syntrophorhabdus*, *Syntrophobacter*, *Methanospurillum*, and *Methanosaeta* (Fig. 4). This is in agreement with a previous study (33), which proposed that acetogens and methanogens form the core of granules and become more abundant with granule growth. The presence of more syntrophic organisms in the granular community is unsurprising, as granulation has long been hypothesized to support increased syntrophy (40). *Geobacter* spp., known electroactive organisms and syntrophic partners of methanogens (41), were also more abundant in the granular communities (Fig. 5). The identification of distinct microbial communities and patterns in community assembly based on granule size confirms our hypothesis that different organisms may dominate and have different functions throughout granule growth and provides evidence for a granulation model based on microbial community succession.

### The active community.

The active community was distinct from the total community in the final takedown (Fig. 5), indicating some redundancy in the total communities. While inactive organisms might have been redundant at sampling, they may have been active at some point during granule growth and involved in granule formation. Their presence in the granule architecture, even if inactive, may be required for the structural integrity of granules.

#### (i) Total versus active communities.

*Geobacter* spp. and *Methanosaeta* spp. were relatively more abundant in the active communities than in the total community and have previously been observed to facilitate direct interspecies electron transport (DIET) (42). DIET between non-surface-attached microorganisms is generally considered to benefit from microbial aggregation (43). DIET was first proposed to occur in methanogenic granules in 2011 (44) and was subsequently confirmed in ethanol-metabolizing granular communities where electron transfer between *Geobacter* spp. and *Mehanosaeta* spp. was confirmed (41). As ethanol was one of the main components of the feedstock here, and both *Geobacter* spp. and *Mehanosaeta* spp. were abundant in the active granular community, it is likely that DIET occurred by this mechanism in the present study and may even have enhanced granulation (Fig. S4).

*Desulfovibrio* spp. were also more abundant in the active than in the total community (Fig. S4). Sulfate-reducing bacteria (SRB), such as *Desulfovibrio* spp., often compete with methanogens in anaerobic environments containing sulfate (45, 46). However, under low-sulfate conditions, such as in the present study, SRBs typically form syntrophic partnerships with hydrogenothrophic methanogens (47–49). This setup was likely in our biomass given the abundant *Desulfovibrio* spp. and hydrogenothrophic methanogen populations in the active granular community.

#### (ii) Comparison of the active community across granule sizes.

Active *Streptococcaceae* were more abundant in XS biomass than in any other size (Fig. S5) in all reactors. In the context of community succession and granulation, *Streptococcaceae* may be primary consumers breaking down the most complex components of the feedstock (lactose) and providing simpler substrates for syntrophic acetogenic/methanogenic populations. *Streptococcaceae* spp. were still present and active in granular communities, but at lower abundances, and could have occupied the granule surface, as was previously hypothesized (37).

The high abundance of active *Thauera* spp. in S granules across all reactors indicates a possible role in initial granule formation (Fig. S5). *Thauera* species are facultative anaerobes often found in aerobic granular bioreactors, where they are thought to play a significant role in EPS production (50–53) and granulation. Aerobic organisms were previously suggested to play a role in anaerobic granulation (54). It may be that *Thauera* spp. are involved in removing residual oxygen in the influent wastewater, developing strict anaerobic environments on granule fragments, and establishing a new EPS matrix. Along with the availability of metabolites from the acidogenic XS fraction, this scenario would provide an ideal environment for new methanogenic granule formation and could potentially be a key stage in community succession and granule growth.

### Mechanism of granule formation.

**FIG 6:**
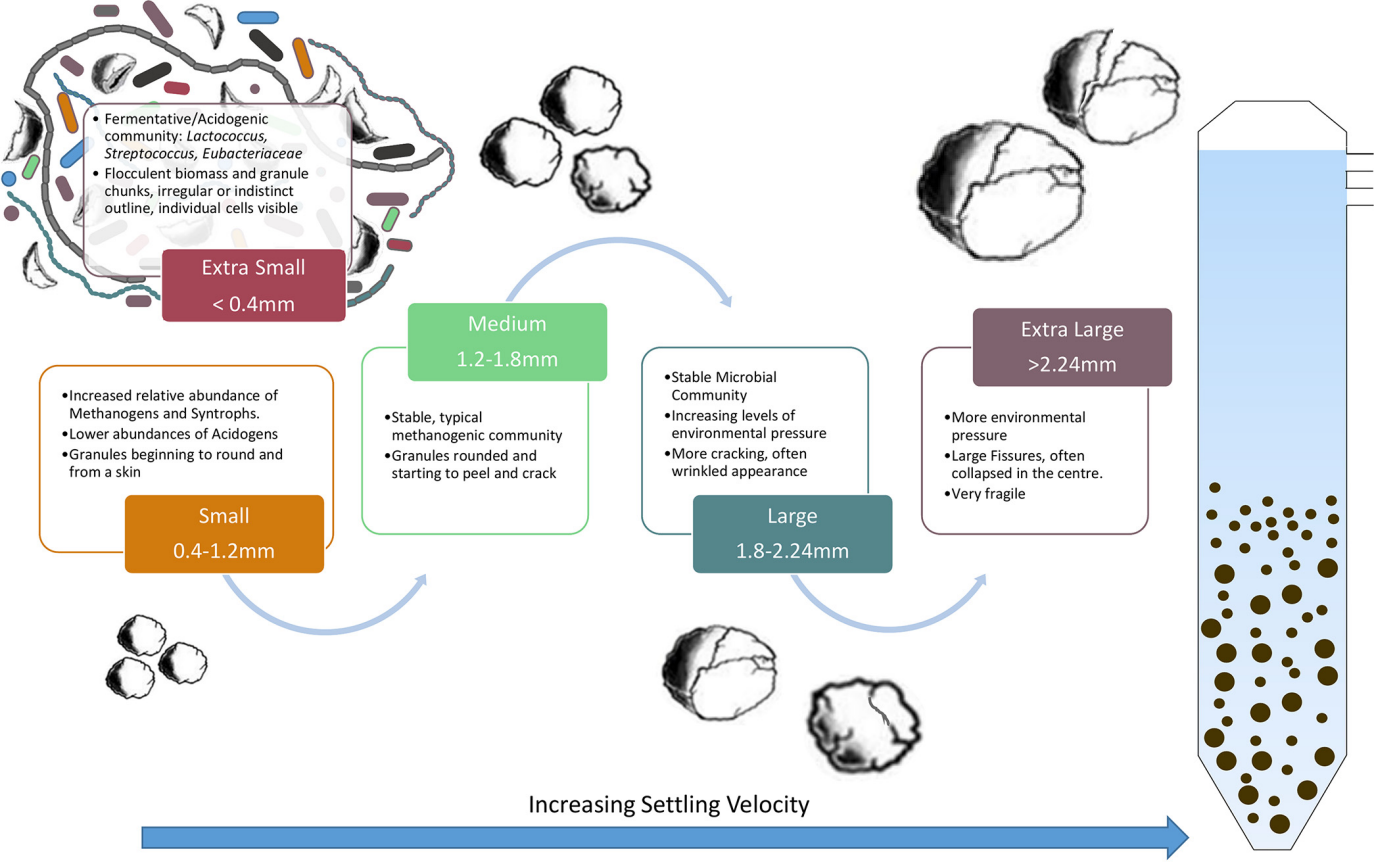
Proposed ecological model for granulation.

It is apparent that the pregranular XS community was distinct from the granular community and may have acted as primary consumers, degrading lactose and providing simpler substrates for methanogenic communities and facilitating granule formation (Fig. 6). The flocculent or filamentous nature of the XS biomass may also have aided in initiating granule formation in a similar manner to that proposed for *Methanosaeta* sp. (39). The initial formation of anaerobic granules and accommodation of strictly anaerobic methanogenic communities may also be facilitated by facultative anaerobes, including *Thauera* spp., which could remove oxygen at a localized scale in S granules and form an EPS matrix. It appears that granulation leads to increased levels of syntrophy, including traditional methanogenesis (acetogens and methanogens), SRBs and hydrogenotrophic methanogens, and DIET-mediated methanogenesis. Once a stable, syntrophic, granule community is formed it, persists during growth until, eventually, increased environmental pressure influences the community. This environmental pressure may be caused by factors such as substrate limitation or gas accumulation inside the granule, leading to granule disintegration. Upon disintegration, this previously isolated community could then contribute to overall diversity in the reactor and facilitate the growth of new granules (Fig. 6). However, further analysis such as the metagenome assembled genomes (MAGs)-based approach (11) would be required to determine if strain-level diversification is indeed occurring inside individual granules. In addition, limitations are always associated with conducting such studies at the laboratory scale (55), and further sampling of full-scale systems would be required to determine if the patterns observed here are universal.

### Conclusion.

Alteration of the initial seed size had only a slight influence on the resultant microbial communities in each reactor, with the operation time and the recovered size causing most of the beta diversity between samples. XS biomass had a significantly different microbial community, both in the seed sludge and at the lab scale, characterized by a higher proportion of acidogenic organisms such as *Streptococcaceae.* It was proposed that this biomass was present as a background flocculent community in the bioreactor or as an outer layer of granules which acted as primary consumers in a community succession model for methanogenic granulation. This flocculent biomass is understudied in granular bioreactors and may have a more important role than previously established. However, more research specifically targeting their role in granulation is required. In contrast to the XS biomass, granular communities had higher proportions of syntrophs and methanogens and resembled a more typical methanogenic community. Therefore, granulation facilitated increased syntrophic interactions. Aerobic organisms such as *Thauera* spp. were most active in S granules, indicating their involvement in the early stages of granule growth, possibly by facilitating the establishment of a strict anaerobic environment or producing an EPS matrix. Environmental pressure increased with granule size, indicating deterministic influences on community assembly in growing granules which may have contributed to overall diversification in the bioreactors.

## MATERIALS AND METHODS

### Source of biomass.

**FIG 7:**
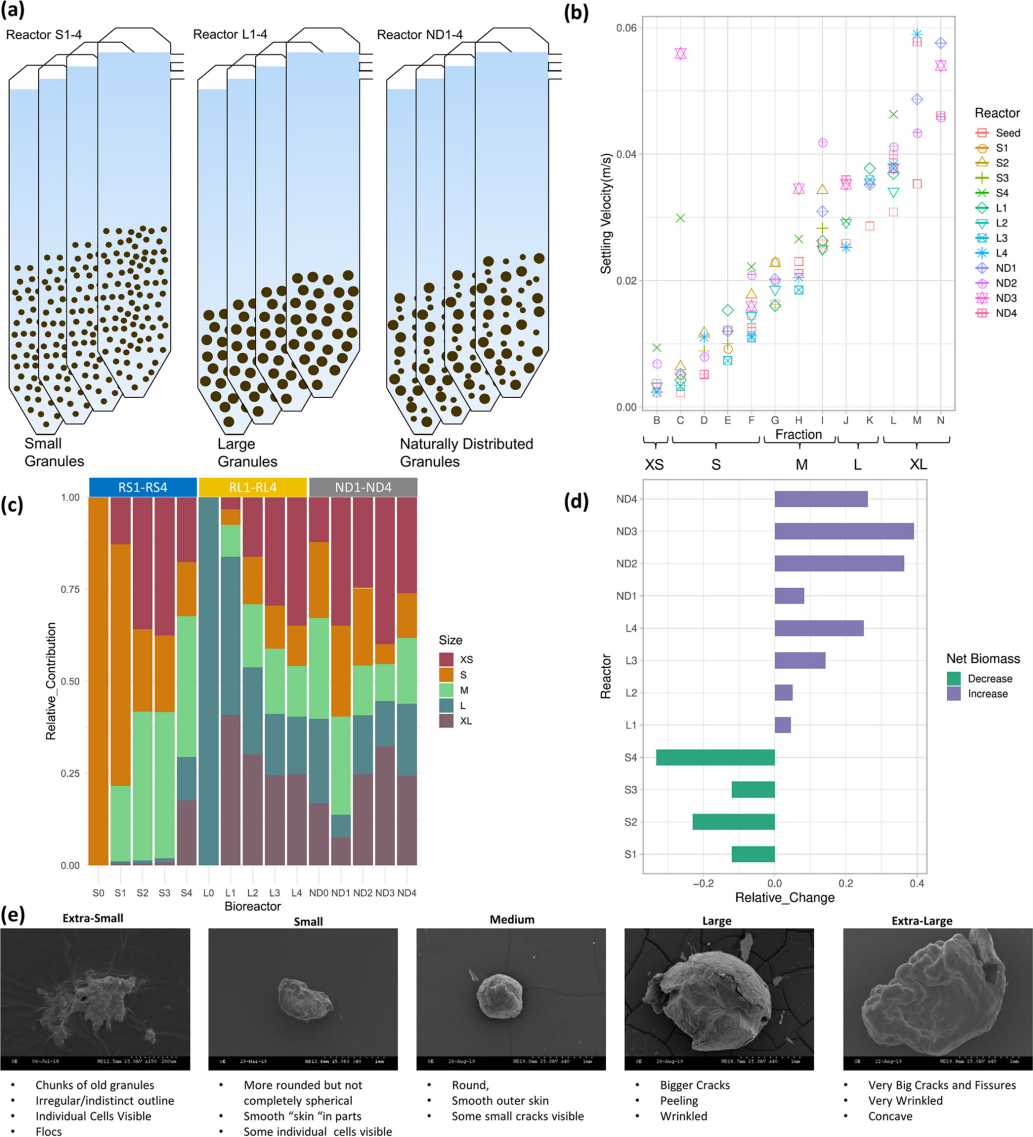
Summary figure. (a) Bioreactor setup; reactors S1 to 4 were seeded with small granules; reactors L1 to 4 were seeded with large granules; reactors ND1 to 4 were seeded with a natural distribution of granules. (b) Settling velocity of granules. (c) Granule size distributions at seeding and takedown of each reactor. (d) Net relative change (%) in biomass volume in each reactor at the time of takedown. (e) Scanning electron microscopy (SEM) images of typical granules from each size bin. All data presented here were reported previously in reference 21.

Granular sludge was sampled from 12 lab-scale (2-liter) expanded granular sludge bed bioreactors treating synthetic wastewater composed of lactose, ethanol, and a volatile fatty acids (VFA) mixture (21). Following initial seeding, all bioreactors were operated identically as reported previously (21). Briefly, bioreactors were seeded in three sets of four replicates, with the only difference being the granule seed size. Initially, biomass sampled from a full-scale internal circulation (IC) bioreactor (650 m^3^) was size-separated into 5 bins, extrasmall (XS) (<0.4 mm), small (S) (0.4 to 1.18 mm), medium (M) (1.18 to 1.8 mm), large (L) (1.8 to 2.24 mm), and extralarge (XL) (>2.24 mm), using a range of grading sieves. Bioreactors were then seeded as follows: four bioreactors (RS1 to RS4) inoculated only with S granules, four bioreactors (RL1 to RL4) inoculated only with L granules, and four bioreactors inoculated with a replete set of naturally distributed (RND1 to RND4), unsieved (ND) granules (Fig. 7). One bioreactor from each of the three sets was sacrificially sampled after 29, 61, 92, and 144 days of operation.

Upon takedown of each sacrificial bioreactor, all sludge was collected, and bioreactor liquor was immediately separated by decanting, to be used as a liquid for initial sieving. A small sample (approximately 50 to 100 ml) of sludge was sieved through 4 stainless steel sieves using only reactor liquor in order to quickly obtain size-separated granular sludge for microbial community analysis. After reactor liquor had passed through the smallest sieve, it was collected and sampled to be used as the XS fraction. All samples were snap-frozen with liquid nitrogen in RNALater and stored at −80°C; the remainder of the sludge was sieved, using tap water, to determine the size distribution.

Temporal changes in granule size distribution, volatile solids content (VS), settling velocity, and biofilm ultrastructure (scanning electron microscopy [SEM]) were monitored and were reported in detail previously (21) and are summarized here (Fig. 7). Size distributions in each of the size-restricted bioreactor sets diversified significantly beyond their original range (Fig. 7) and tended to revert back to a distribution similar to that of the natural distribution in the initial ND seed sludge. XS biomass was generally flocculent and, upon examination, contained chunks and pieces of larger granules, indicating that this fraction was made up of both flocculent growth and pieces of broken granules.

Reactor performance was monitored in terms of soluble chemical oxygen demand (sCOD) removal. and methane production and was reported in detail previously (21). Biomass washout was assessed by measuring particulate COD (pCOD) in the effluent. In terms of sCOD removal, RND1-4 performed slightly better than RS1-4 and RL1-4 (21). RND1-4 also experienced significantly lower levels of biomass washout (Fig. 7) and generally performed in a more stable manner, particularly during the initial 50 days of operation (21). RS4 experienced a slight drop in performance during the last month of operation, with slightly poorer sCOD removal efficiency, which was attributed to prolonged biomass washout (Fig. 7).

### DNA extraction, cDNA synthesis, PCR, and sequencing.

Genomic DNA and RNA were coextracted according to a previously described protocol (56). Snap-frozen samples were crushed using a glass rod in a microcentrifuge tube to obtain a homogeneous mixture. Cells were lysed by beat beating in a 1% cetyltrimethylammonium bromide (CTAB) lysis buffer, followed by phenol-chloroform coextraction. DNA and RNA concentrations were quantified using a Qubit fluorometer (Invitrogen, Carlsbad, CA, USA).

Samples from the final takedown (RS4, RL4, and RND4) were used for analysis of the active community by sequencing cDNA which was reverse-transcribed from 16S rRNA gene transcripts. Prior to cDNA synthesis, contaminating DNA was removed from RNA samples using the TurboDNase kit (AMBION-Invitrogen, Carlsbad, CA, USA) following the manufacturer’s instructions. Complete removal of DNA was confirmed by PCR using the primer pair 515F and 806R. cDNA synthesis was carried out with the Superscript IV (SSIV) reverse transcriptase kit (Thermo Fisher, Waltham, MA, USA) according to the manufacturer’s instructions.

PCR amplification of DNA and cDNA was performed using the KAPA HiFi HotStart ReadyMix and 515F 806R primer pair (57) at a final concentration of 0.2 μM, with 12.5 ng of template. PCR cycles were as follows: initial denaturation was performed for 3 min at 95°C, followed by 25 cycles of denaturation at 95°C for 30 s, annealing at 55°C for 30 s, and extension at 72°C for 30 s. Library preparation and sequencing were carried out by The Foundation for the Promotion of Health and Biomedical Research of Valencia Region (FISABIO) (Valencia, Spain). Then, 300-bp paired-end sequences were generated using Illumina V3 chemistry on the Illumina MiSeq platform.

### Bioinformatic analysis.

Amplicon sequencing variants (ASVs) were constructed using the QIIME 2 workflow with the DADA2 algorithm (58). All code for bioinformatic processing of sequences has been made available (https://github.com/umerijaz/tutorials/blob/master/qiime2_tutorial.md). Summary statistics for sample reads were as follows: 1st quantile, 76,758; median, 114,026; mean, 111,824; 3rd quantile, 134,002; maximum, 260,003. The number of reads assigned to ASVs per sample has been included in Table S2. In the final analysis, 5,285 clean ASVs were extracted for *n* = 240 samples on which different multivariate statistical analyses were performed using R software. The details of statistical analyses as well as software and R packages used are provided in the File S1.

### Data availability.

The raw sequencing data have been deposited in the Sequence Read Archive (SRA) hosted by the National Center for Biotechnology Information (NCBI) under the BioProject accession number PRJNA757966.
