# Supplementary material for: A Distinct, Flocculent, Acidogenic Microbial Community Accompanies Methanogenic Granules in Anaerobic Digesters
Source: Microbiol Spectr. 2021 Nov 10;9(3):e00784-21. doi: 10.1128/Spectrum.00784-21 (PMC8579839; doi:10.1128/Spectrum.00784-21)
Supplement: SUPPLEMENTAL FILE 2 — Supplemental material. Download SPECTRUM00784-21_Supp_2_seq12.pdf, PDF file, 6.6 MB [file spectrum00784-21_supp_2_seq12.pdf]

SUPPLEMENTAL MATERIAL

A Distinct, Flocculent, Acidogenic Microbial Community Accompanies Methanogenic Granules in Anaerobic Digesters

Simon Mills[a], Anna Christine Trego[a], Piet N.L. Lens[b], Umer Zeeshan Ijaz[c#] and Gavin Collins[a,c,d,#]

[a]Microbial Communities Laboratory, School of Natural Sciences, National University of Ireland Galway, University Road, Galway, H91 TK33, Ireland

[b]IETSBIO3 Laboratory, National University of Ireland, Galway, University Road, Galway, H91 TK33, Ireland

[c]Infrastructure and Environment, School of Engineering, The University of Glasgow, Oakfield Avenue, Glasgow G12 8LT, United Kingdom

[d]Ryan Institute, National University of Ireland Galway, University Road, Galway, H91 TK33, Ireland

[#]Joint Corresponding authors:

Gavin Collins, Ph.D.,	Umer Zeeshan Ijaz, Ph.D.
T: +353 (0) 91 49 31 63	+44 (0) 141 330 6458
E: gavin.collins@nuigalway.ie	umer.ijaz@glasgow.ac.uk

Running title: Ecology of Methanogenic Granulation

Supplemental Materials and Methods

Statistical Analysis

The vegan package (Oksanen et al., 2015) was used to assess alpha and beta diversity. Alpha diversity was assessed using the following indices: (i) rarefied richness – an estimation of the total number of ASVs in a rarefied sample (to minimum library size); (ii) Shannon entropy – a commonly used diversity index; (iii) Pilou eveness – a comparison of the actual diversity values to the highest possible diversity value, constrained from 0 to 1.0, whereby lower values indicate more variation in abundance between different ASVs in each group. Beta diversity was assessed with Principal Coordinate Analysis (PCoA). ASVs were plotted with (i) the Bray-Curtis distance metric which considers only ASV abundance counts. Multivariate homogeneity of sample variability was assessed using Vegan's betadisper() function, this handles distances between objects and group centroids by reducing the original distances to principal coordinates and reporting significances based on Analysis of variance (ANOVA). ANOVA was carried out with Vegan's Adonis() function on distance matrices. This function, referred to as PERMANOVA, fits linear models to distance matrices contingent upon the explanatory variables (meta data) for a given study to assess how much of the variability in microbial community structure they can explain. Local Contribution to Beta Diversity (LCBD) analysis (Legendre and De Cáceres, 2013) was performed with the LCBD.comp() function from the adespatial package (Dray et al., 2016). Bray-Curtis, Unifrac and Weighted Unifrac, distances were used. LCBD assesses how far the microbial community structure of one sample is from the average of all the samples. Phylogenetic distances within each sample were assessed by calculating the nearest taxa index (NTI) and net relatedness index (NRI). NTI was calculated with mntd() and ses.mntd(), and the mean phylogenetic diversity (MPD) and NRI were calculated using mpd() and ses.mpd() functions from the picante package (Kembel et al., 2010). NTI and NRI represent the negatives of the output from ses.mntd() and ses.mpd(), respectively. Additionally, they quantify the number of standard deviations that separate the observed values from the mean of the null distribution

(999 randomisation using null.model-'richness' in the ses.mntd() and ses.mpd() functions and only considering taxa as either present or absent regardless of their relative abundance). A positive NTI value indicates that species co-occur with more closely related species than would be expected by chance, whereas negative values suggesting otherwise. NTI measures tip-level divergences (putting more emphasis on terminal clades and is akin to "local" clustering) in phylogeny while NRI measures deeper divergences (akin to "global" clustering or "clumpedness"). For both NTI and NRI, values > +2 indicate strong environmental pressure, and values < -2 indicate strong competition among species as the driver of community structure

Sparse Projection to Latent Structure – Discriminant Analysis (sPLS-DA) was carried out using the MixOmics package (Rohart et al., 2017). Here, artificial latent components were constructed for predicted variables (ASVs) and response variables (sample groups) by factoring these matrices into scores and loading vectors in a new space to achieve a maximum covariance between the scores of these two matrices. Loading vectors (with piece-wise coefficient for each ASV) were constructed so that the coefficients indicate the importance of each variable to define the component. Non-zero coefficients for the loading vectors indicate ASVs which are significantly different between the categories and are deemed discriminants. The initial ASV table was prefiltered by removing 1% of ASVs with low counts according to the author's recommendations given at http://mixomics.org/mixmc/pre-processing/. Following this, the ASV table was normalised using Total Sum Scaling (TSS) on the ASVs and then Centered Log Ratio (CLR) (in conjunction these are referred to as TSS+CLR normalization) before applying the splsda() function. The perf.plsda() and tune.splsda() functions were initially used to predict the number of latent components (associated loading vectors) and the number of discriminants by initializing the perf.plsda() procedure with the total number components to be the number of groups used in the study. Then, the first two components were retained as the classification error rates were minimum for these using the centroid distance matrix in the

procedure. The tune.splsda() function was then initialized with two components and using leave-one-out cross-validation.

Additionally, a study-wise comparison of reactors (RS1-4, RL1-4, and RND1-4) was done to find discriminating taxa between recovered granule sizes (XS, S, M, L, and XL). This was done using the Multivariate Integration (MINT) algorithm (Rohart et al., 2017). The algorithm is an extension of the multi-group Projection to Latent Structure (mgPLS), similar to sPLS-DA and it attempts to find a common projection space across all studies (reactors as above), defined on a small subset of discriminative variables that consistently discriminate the outcome classes (granule sizes). In MINT, for every reactor set we have combined four datasets where all these datasets share the taxa whilst the number of samples differ. Similar to sPLS-DA, we have performed prefilteration of 1% of the lowest abundant taxa followed by TSS+CLR normalization. Differential taxa, identified by MINT analysis were visualized using differential heat trees (Foster et al., 2017) which compared the relative abundance of these taxa (using Wilcoxin p-value test) between different sample groups. The authors have applied these methods before with further details given in (Trego et al., 2020).

We performed subset regression against different microbiome metrics (Supplementary Tables 3-9) by testing all possible combination of the meta data in this study, and then selecting the best model according to some statistical criteria, with recommendations given in Kassambara (2018) with code available at http://www.sthda.com/english/articles/37-model-selection-essentials-in-r/155-best-subsets-regression-essentials-in-r/. The R function regubsets() from the leaps package (Lumley and Miller, 2009) was used to identify different best models of different sizes, by specifying the option nvmax, set to the maximum number of predictors to incorporate the model. Having obtained the best possible subsets, the k-fold cross-validation consisting of first dividing the data into k subsets. Each subset (10%) served successively as test data set and the remaining subset (90%) as training data. The average cross-validation error is then computed as the model prediction error. This was all done using a custom function utilising R's train() function from the caret package (Kuhn, 2005).

Finally R's tab_model() function from sjPlot package (Lüdecke, 2019) was used to obtain the statistics for each model.

Supplemental Figures and Tables

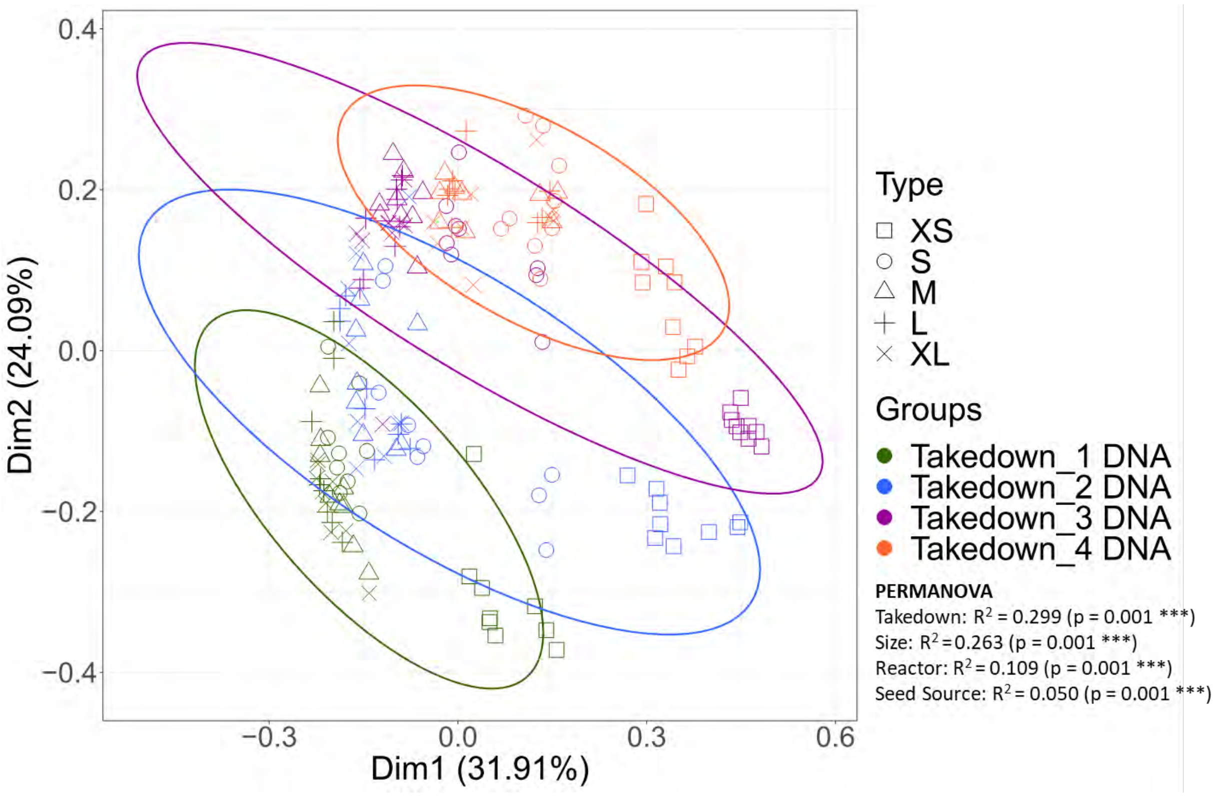

Figure. S1 Principle Component Analysis (PCOA) of total microbial community, using the Bray-Curtis distance metric, where ellipses were drawn using 95% confidence intervals based on standard deviation. Samples were grouped by takedown (Colour) and shapes represent biomass size.

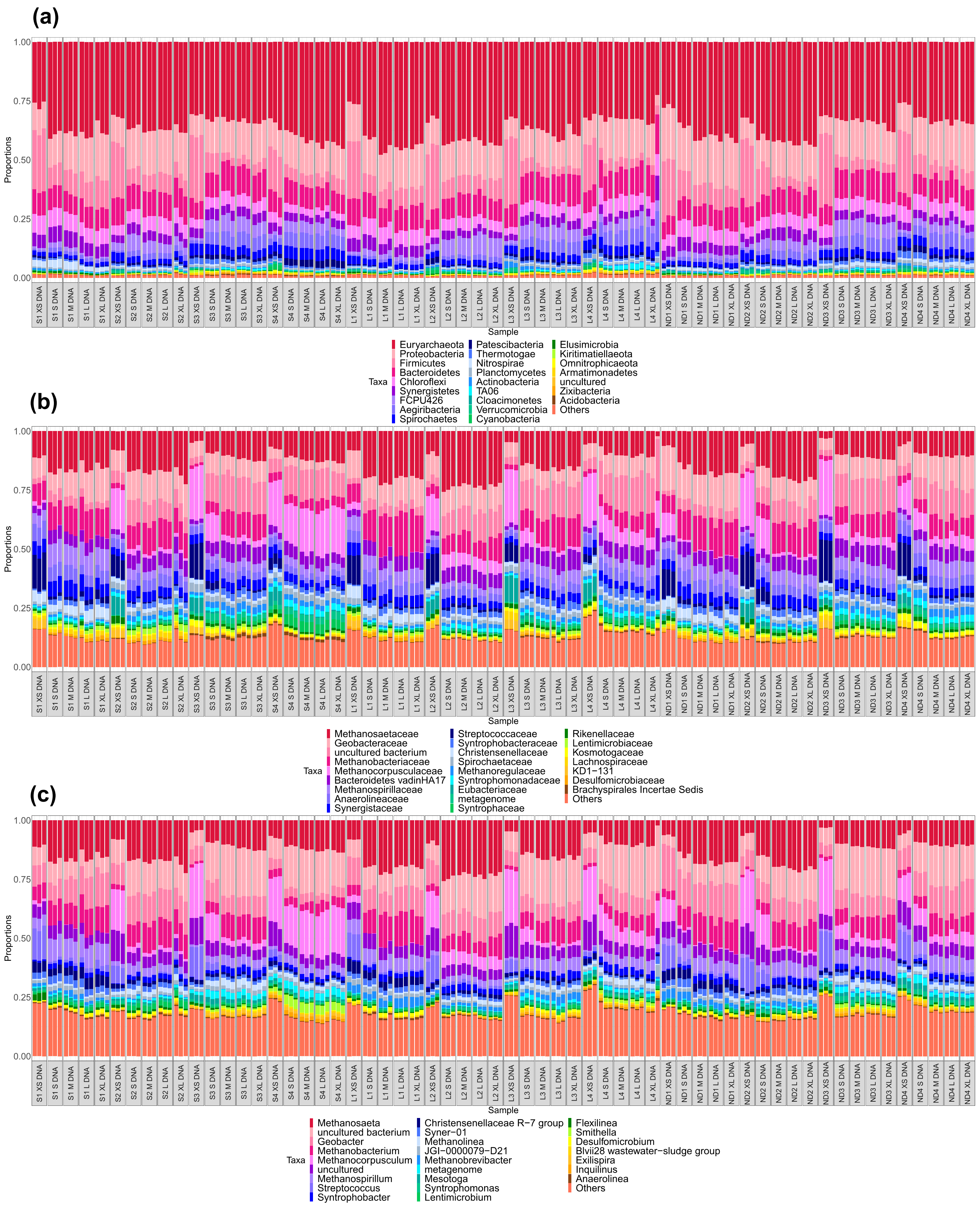

Figure. S2. Bar charts depicting the top 25 most abundant taxa at a) Phylum level b) Family and c) Genus, where others represents taxa outside of the top 25 most abundant organisms.

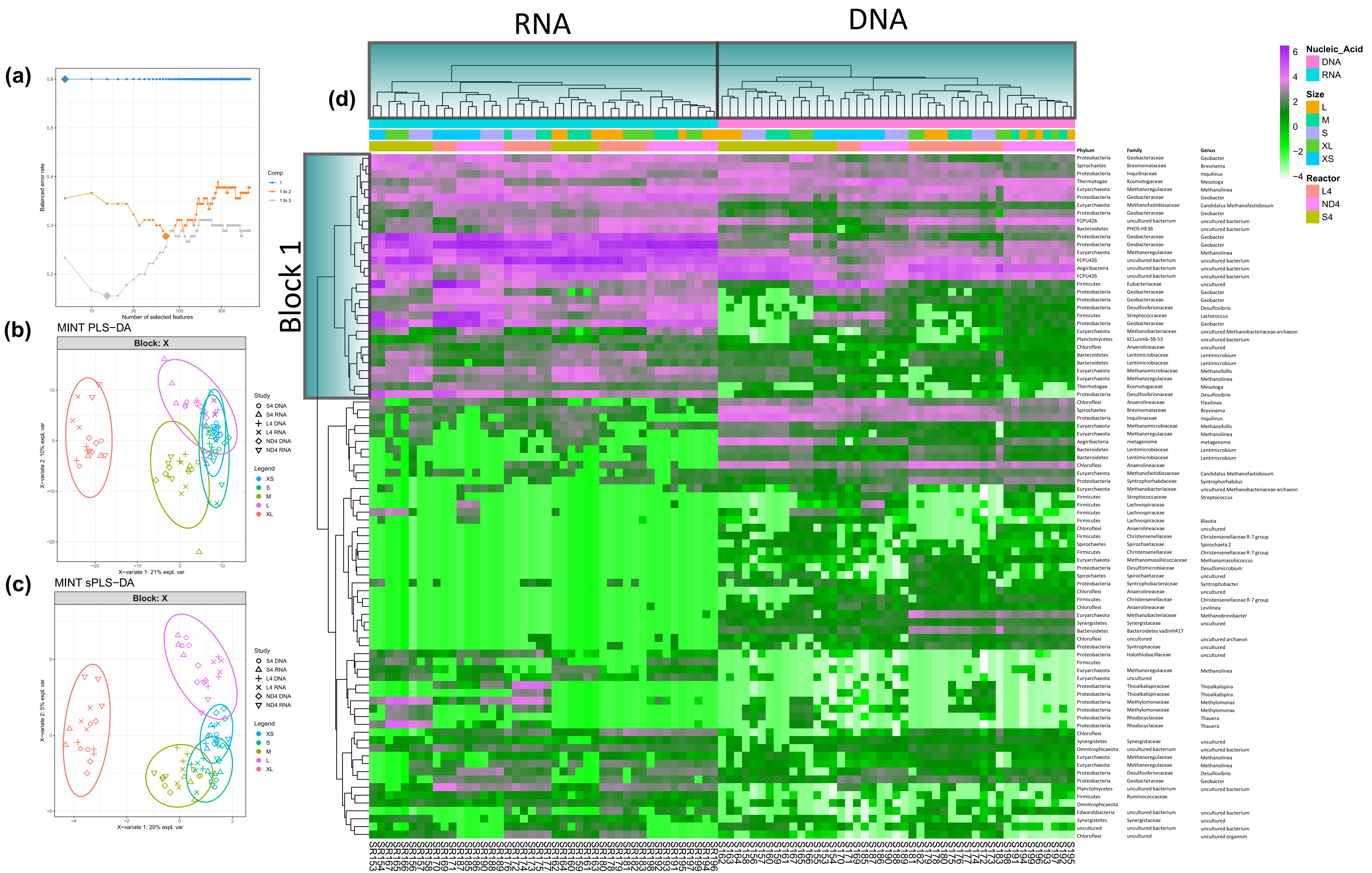

Figure. S3 (a) Classification error rates over the components and the numbers of optimal features (genera) in each component, included in the model, chosen by the lowest error rates which are denoted by diamonds. (b) Ordination of whole ASV table (c)Ordination of discriminant ASVs only (d) Heatmap depicting discriminant Genera. Rows and columns are ordered according to hierarchical (average linking) clustering to identify clusters of Genera among groups

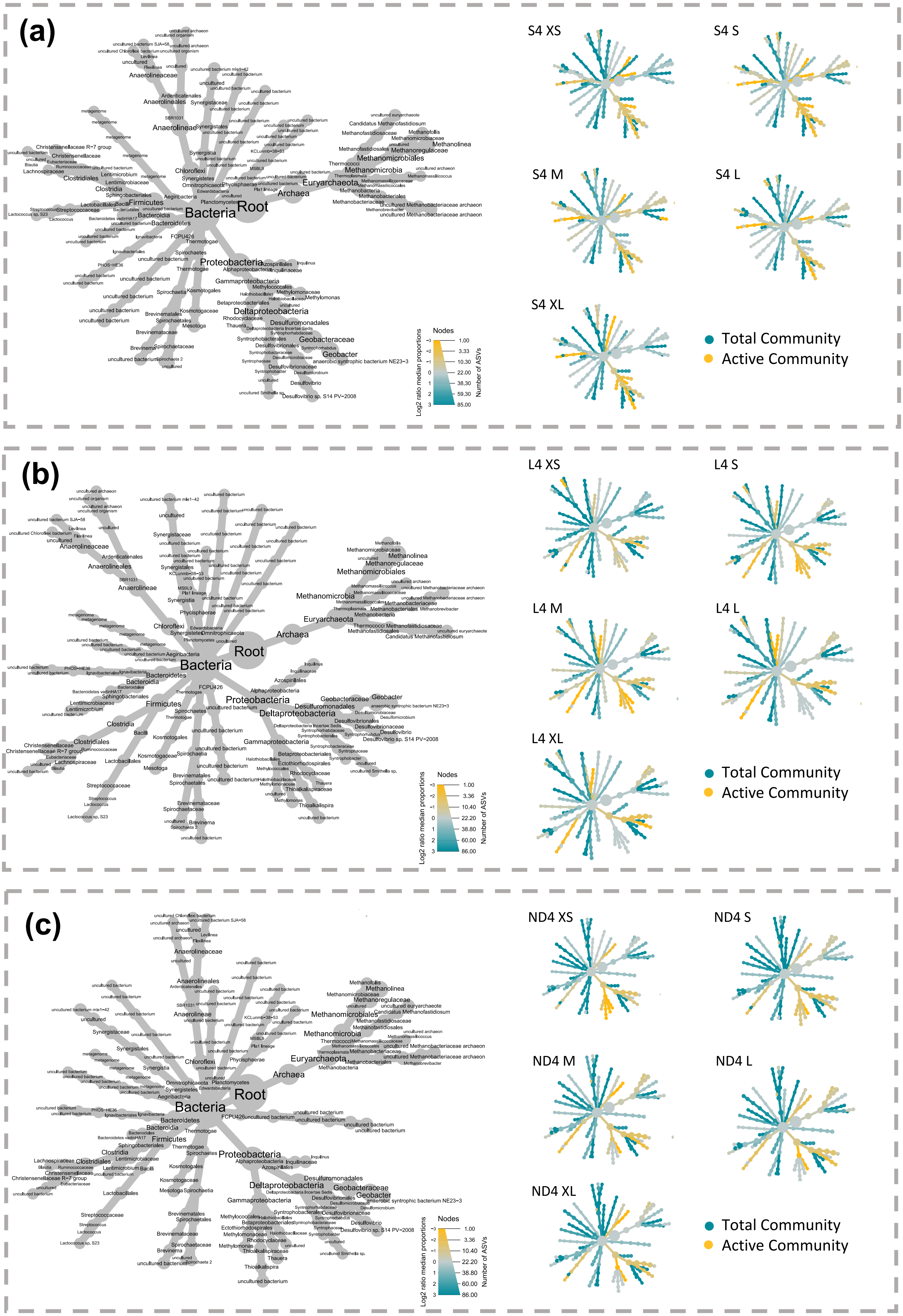

Figure. S4 Heat Trees depicting differential abundances of discriminant ASVs (identified by MINT analysis (Fig S5)) in the total (DNA) and active (RNA) community of all granule sizes from RS4, RL4 and RND4. The circle size and the colour intensity reflect the species abundance and the log2 median proportion between the two groups respectively

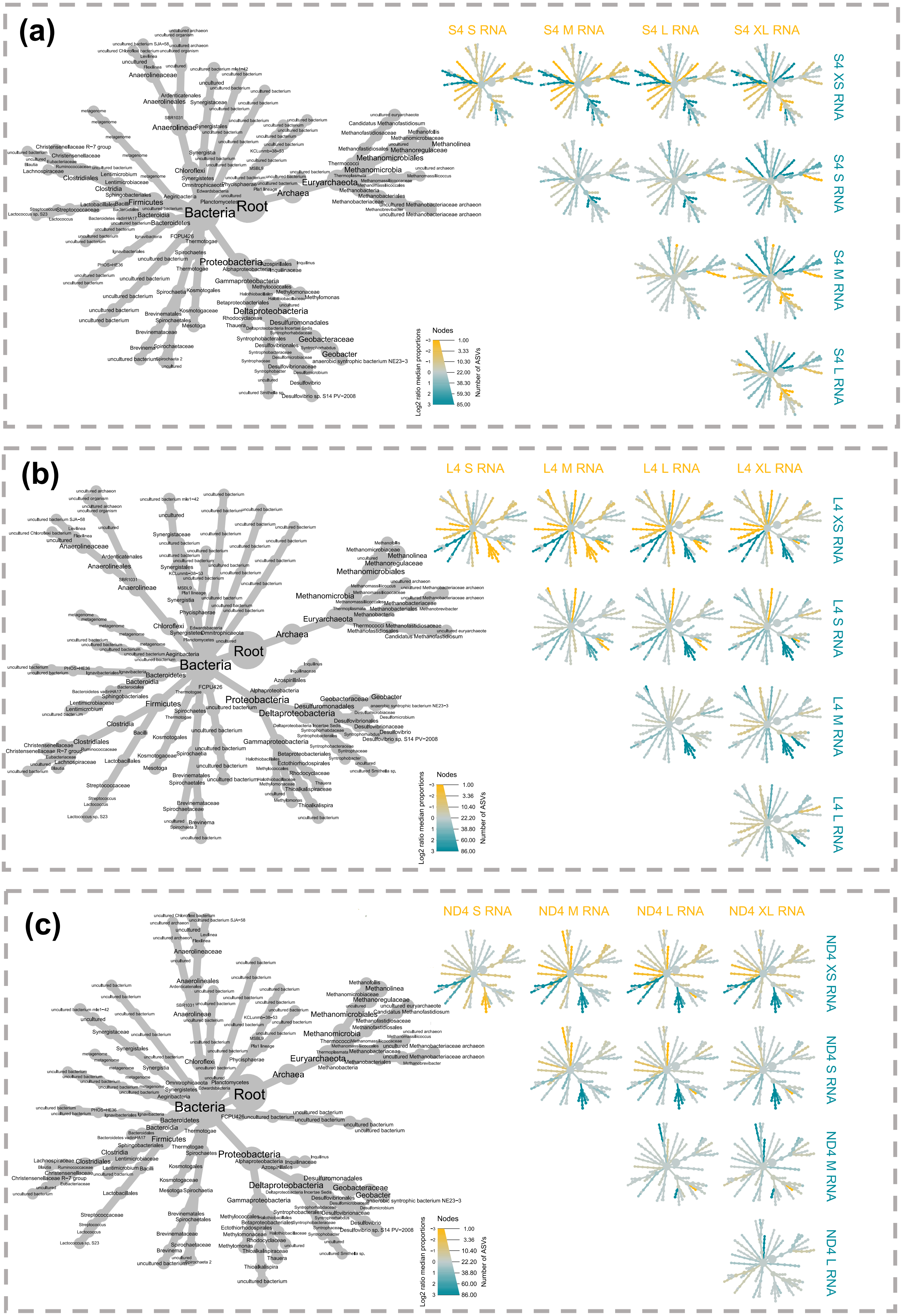

Figure. S5 Heat Trees depicting differential abundances of discriminant ASVs (identified by MINT analysis (Fig S5)) in the active (RNA) community of all granule sizes from RS4, RL4 and RND4. The circle size and the colour intensity reflect the species abundance and the log2 median proportion between the two groups respectively

Table S1 Bioreactor performance data used in Subset Regression Analysis

	Bioreactor Performance Metrics											
**Day**	**S1**	**S2**	**S3**	**S4**	**L1**	**L2**	**L3**	**L4**	**ND1**	**ND2**	**ND3**	**ND4**
sCOD (%Removal)	98% +/- 0.93%	98% +/- 0.72%	98% +/- 0.95%	94% +/- 1.07%	97% +/- 1.25%	97% +/- 3.14%	99% +/- 0.25%	98% +/- 0.55%	98% +/- 1.51%	98% +/- 1.22%	97% +/- 0.35%	99% +/- 0.58%
pCOD (mgCOD/l)	2178 +/- 1369	1673 +/- 972	1512 +/- 670	1551 +/- 229	1202 +/- 530	1685 +/- 807	1889 +/- 643	1141 +/- 134	1454 +/- 1212	1455 +/- 439	1145 +/- 207	1118 +/- 468
Methane Production (mlCH_4/h)	294 +/- 96	323 +/- 85	277 +/- 75	361 +/- 41	343 +/- 153	319 +/- 58	318 +/- 68	348 +/- 19	354 +/- 149	364 +/- 49	344 +/- 56	324 +/- 28

Reported values averaged over final 20/21 days of reactor operation +/- standard deviations.

Table S2 Number of reads assigned to ASVs per sample

Sample	Code	Reads to ASVs	Sample	Code	Reads to ASVs
sm_1	T0_XS_1	53147	sm_123	L3_XS_1	125206
sm_2	T0_XS_2	62479	sm_124	L3_XS_2	136707
sm_3	T0_XS_3	71049	sm_125	L3_XS_3	122326
sm_4	T0_S_1	61660	sm_126	L3_S_1	130829
sm_5	T0_S_2	62018	sm_127	L3_S_2	147396
sm_6	T0_S_3	48821	sm_128	L3_S_3	132739
sm_7	T0_M_1	65709	sm_129	L3_M_1	132524
sm_8	T0_M_2	65582	sm_130	L3_M_2	140030
sm_9	T0_M_3	59893	sm_131	L3_M_3	178072
sm_10	T0_L_1	66105	sm_132	L3_L_1	131000
sm_11	T0_L_2	58614	sm_133	L3_L_2	97737
sm_12	T0_L_3	62599	sm_134	L3_L_3	99793
sm_13	T0_XL_1	54880	sm_135	L3_XL_1	61217
sm_14	T0_XL_2	60392	sm_136	L3_XL_2	123862
sm_15	T0_XL_3	73537	sm_137	L3_XL_3	121361
sm_16	S1_XS_1	68683	sm_138	ND3_XS_1	106692
sm_17	S1_XS_2	81871	sm_139	ND3_XS_2	126917
sm_18	S1_XS_3	82348	sm_140	ND3_XS_3	117815
sm_19	S1_S_1	122693	sm_141	ND3_S_1	139397
sm_20	S1_S_2	67733	sm_142	ND3_S_2	183540
sm_21	S1_S_3	61273	sm_143	ND3_S_3	126689
sm_22	S1_M_1	70865	sm_144	ND3_M_1	129269
sm_23	S1_M_2	52433	sm_145	ND3_M_2	147065
sm_24	S1_M_3	66041	sm_146	ND3_M_3	101664
sm_25	S1_L_1	143533	sm_147	ND3_L_1	124666
sm_26	S1_L_2	73416	sm_148	ND3_L_2	141050
sm_27	S1_L_3	77086	sm_149	ND3_L_3	160432
sm_28	S1_XL_1	49628	sm_150	ND3_XL_1	130578
sm_29	S1_XL_2	73774	sm_151	ND3_XL_2	126776
sm_30	S1_XL_3	61379	sm_152	ND3_XL_3	157711
sm_31	L1_XS_1	73606	sm_153	S4_XS_1	159102
sm_32	L1_XS_2	64714	sm_154	S4_XS_2	260003
sm_33	L1_XS_3	162566	sm_155	S4_XS_3	173957
sm_34	L1_S_1	74484	sm_156	S4_S_1	205617
sm_35	L1_S_2	83614	sm_157	S4_S_2	131641
sm_36	L1_S_3	69837	sm_158	S4_S_3	112843
sm_37	L1_M_1	50555	sm_159	S4_M_1	108953
sm_38	L1_M_2	63625	sm_160	S4_M_2	127498
sm_39	L1_M_3	62053	sm_161	S4_M_3	129503
sm_40	L1_L_1	54861	sm_162	S4_L_1	114053
sm_41	L1_L_2	58737	sm_163	S4_L_2	118245

sm_42	L1_L_3	94004	sm_164	S4_L_3	132309
sm_43	L1_XL_1	57047	sm_165	S4_XL_1	134522
sm_44	L1_XL_2	62871	sm_166	S4_XL_2	140405
sm_45	L1_XL_3	66211	sm_167	S4_XL_3	135394
sm_46	ND1_XS_1	61186	sm_169	L4_XS_1	135033
sm_47	MD1_XS_2	70045	sm_170	L4_XS_2	109640
sm_48	ND1_XS_3	82404	sm_171	L4_XS_3	196418
sm_49	ND1_S_1	104268	sm_172	L4_S_1	249492
sm_50	ND1_S_2	90817	sm_173	L4_S_2	122066
sm_51	ND1_S_3	137120	sm_174	L4_S_3	112683
sm_52	ND1_M_1	89052	sm_175	L4_M_1	111776
sm_53	ND1_M_2	119604	sm_176	L4_M_2	135442
sm_54	ND1_M_3	84515	sm_177	L4_M_3	130716
sm_55	ND1_L_1	171520	sm_178	L4_L_1	129754
sm_56	ND1_L_2	78419	sm_179	L4_L_2	108881
sm_57	ND1_L_3	69656	sm_180	L4_L_3	135373
sm_58	ND1_XL_1	83559	sm_181	L4_XL_1	118288
sm_59	ND1_XL_2	80378	sm_182	L4_XL_2	109025
sm_60	ND1_XL_3	72401	sm_183	L4_XL_3	225112
sm_61	S2_XS_1	72981	sm_185	ND4_XS_1	219883
sm_62	S2_XS_2	81781	sm_186	ND4_XS_2	121722
sm_63	S2_XS_3	87643	sm_187	ND4_XS_3	139466
sm_64	S2_S_1	85685	sm_188	ND4_S_1	209526
sm_65	S2_S_2	144162	sm_189	ND4_S_2	131266
sm_66	S2_S_3	154017	sm_190	ND4_S_3	115941
sm_67	S2_M_1	84018	sm_191	ND4_M_1	125973
sm_68	S2_M_2	73628	sm_192	ND4_M_2	143416
sm_69	S2_M_3	129559	sm_193	ND4_M_3	162802
sm_70	S2_L_1	95964	sm_194	ND4_L_1	223256
sm_71	S2_L_2	87555	sm_195	ND4_L_2	149731
sm_72	S2_L_3	71331	sm_196	ND4_L_3	154765
sm_73	S2_XL_1	243498	sm_197	ND4_XL_1	163133
sm_74	S2_XL_2	80969	sm_198	ND4_XL_2	110603
sm_75	S2_XL_3	66292	sm_199	ND4_XL_3	141717
sm_76	L2_XS_1	79932	sm_R153	S4_XS_1	122171
sm_77	L2_XS_2	87580	sm_R154	S4_XS_2	111632
sm_78	L2_XS_3	126543	sm_R155	S4_XS_3	115
sm_79	L2_S_1	67963	sm_R156	S4_S_1	255040
sm_80	L2_S_2	77239	sm_R157	S4_S_2	108989
sm_81	L2_S_3	66660	sm_R158	S4_S_3	101610
sm_82	L2_M_1	131072	sm_R159	S4_M_1	100037
sm_83	L2_M_2	72088	sm_R160	S4_M_2	125194
sm_84	L2_M_3	67567	sm_R161	S4_M_3	101745
sm_85	L2_L_1	68255	sm_R162	S4_L_1	142466
sm_86	L2_L_2	71785	sm_R163	S4_L_2	104581

sm_87	L2_L_3	75773	sm_R164	S4_L_3	155780
sm_88	L2_XL_1	78411	sm_R165	S4_XL_1	115561
sm_89	L2_XL_2	80152	sm_R166	S4_XL_2	79953
sm_90	L2_XL_3	67226	sm_R167	S4_XL_3	98577
sm_91	ND2_XS_1	66976	sm_R169	L4_XS_1	114986
sm_92	ND2_XS_2	71217	sm_R170	L4_XS_2	133828
sm_93	ND2_XS_3	63009	sm_R171	L4_XS_3	121271
sm_94	ND2_S_1	82436	sm_R172	L4_S_1	119533
sm_95	ND2_S_2	51616	sm_R173	L4_S_2	140407
sm_96	ND2_S_3	68726	sm_R174	L4_S_3	131149
sm_97	ND2_M_1	110627	sm_R175	L4_M_1	149430
sm_98	ND2_M_2	85974	sm_R176	L4_M_2	136289
sm_99	ND2_M_3	151564	sm_R177	L4_M_3	145117
sm_100	ND2_L_1	126939	sm_R178	L4_L_1	126040
sm_101	ND2_L_2	113999	sm_R179	L4_L_2	107387
sm_102	ND2_L_3	122851	sm_R180	L4_L_3	116584
sm_103	ND2_XL_1	111504	sm_R181	L4_XL_1	115640
sm_104	ND2_XL_2	161029	sm_R182	L4_XL_2	122016
sm_105	ND2_XL3	136766	sm_R183	L4_XL_3	123625
sm_106	S3_XS_1	118785	sm_R185	ND4_XS_1	105312
sm_107	S3_XS_2	111079	sm_R186	ND4_XS_2	115235
sm_108	S3_XS_3	146556	sm_R187	ND4_XS_3	152298
sm_109	S3_S_1	133201	sm_R188	ND4_S_1	123737
sm_110	S3_S_2	91031	sm_R189	ND4_S_2	104320
sm_111	S3_S_3	127526	sm_R190	ND4_S_3	124872
sm_113	S3_M_1	124679	sm_R191	ND4_M_1	141014
sm_114	S3_M_2	157919	sm_R192	ND4_M_2	110941
sm_115	S3_M_3	136531	sm_R193	ND4_M_3	101149
sm_116	S3_L_1	112162	sm_R194	ND4_L_1	133694
sm_117	S3_L_2	155110	sm_R195	ND4_L_2	111584
sm_118	S3_L_3	148939	sm_R196	ND4_L_3	128541
sm_119	S3_XL_1	136431	sm_R197	ND4_XL_1	125335
sm_120	S3_XL_2	136635	sm_R198	ND4_XL_2	103925
sm_121	S3_XL_3	133149	sm_R199	ND4_XL_3	155722

Appendix 1 Subset Regression Analysis

Subset Regression Analysis – Species Richness

Model Number	Model	Cross-validation Errors
M3	Richness ~ Status_S + ml_Methane_per_Hr + pCOD_Washout	147.68487
M2	Richness ~ ml_Methane_per_Hr + pCOD_Washout	148.12412
M4	Richness ~ Status_S + ml_Methane_per_Hr + sCOD_Removal + pCOD_Washout	148.82213
M1	Richness ~ pCOD_Washout	150.26180
M5	Richness ~ Status_XS + Status_S + ml_Methane_per_Hr + sCOD_Removal + pCOD_Washout	150.71360
M6	Richness ~ Status_XS + Status_S + Status_XL + ml_Methane_per_Hr + sCOD_Removal + pCOD_Washout	152.61213
M7	Richness ~ Status_XS + Status_S + Status_M + Status_L + ml_Methane_per_Hr + sCOD_Removal + pCOD_Washout	153.60069

	M1-Richness							
Predictors	*Estimates*	*std. Error*	*std. Beta*	*standardized std. Error*	*CI*	*standardized CI*	*Statistic*	*df*
(Intercept)	862.63402 ***	55.05410	0.00000	0.07072	753.99131 – 971.27673	-0.13956 – 0.13956	0.00000	178.00000
pCOD_Washout	-0.16396 ***	0.03593	-0.32366	0.07092	-0.23485 – -0.09306	-0.46361 – -0.18371	-4.56376	178.00000
Observations	180							
R^2 / R^2 adjusted	0.105 / 0.100							

* *p<0.05*   ** *p<0.01*   *** *p<0.001*

	M2-Richness							
Predictors	*Estimates*	*std. Error*	*std. Beta*	*standardized std. Error*	*CI*	*standardized CI*	*Statistic*	*df*
(Intercept)	1335.50011 ***	201.99576	0.00000	0.06977	936.87012 – 1734.13011	-0.13768 – 0.13768	0.00000	177.00000
ml_Methane_per_Hr	-1.20948 *	0.49763	-0.19518	0.08031	-2.19154 – -0.22742	-0.35366 – -0.03670	-2.43047	177.00000
pCOD_Washout	-0.21250 ***	0.04068	-0.41949	0.08031	-0.29278 – -0.13222	-0.57797 – -0.26100	-5.22356	177.00000
Observations	180							
R^2 / R^2 adjusted	0.134 / 0.124							

* *p<0.05   ** p<0.01   *** p<0.001*

	**M3-Richness**							
Predictors	*Estimates*	*std. Error*	*std. Beta*	*standardized std. Error*	*CI*	*standardized CI*	*Statistic*	*df*
(Intercept)	1328.90310 ***	201.83069	0.00000	0.06968	930.58330 – 1727.22290	-0.13752 – 0.13752	0.00000	176.00000
Status_S	32.98506	27.65251	0.08335	0.06988	-21.58812 – 87.55825	-0.05455 – 0.22126	1.19284	176.00000
ml_Methane_per_Hr	-1.20948 *	0.49704	-0.19518	0.08021	-2.19041 – -0.22856	-0.35348 – -0.03688	-2.43337	176.00000
pCOD_Washout	-0.21250 ***	0.04063	-0.41949	0.08021	-0.29269 – -0.13231	-0.57778 – -0.26119	-5.22979	176.00000
Observations	180							
R^2 / R^2 adjusted	0.141 / 0.126							

* p<0.05   ** p<0.01   *** p<0.001

	**M4-Richness**							
Predictors	*Estimates*	*std. Error*	*std. Beta*	*standardized std. Error*	*CI*	*standardized CI*	*Statistic*	*df*
(Intercept)	2092.54539	1158.28837	0.00000	0.06979	-193.46696 – 4378.55774	-0.13774 – 0.13774	0.00000	175.00000
Status_S	32.98506	27.69595	0.08335	0.06999	-21.67602 – 87.64614	-0.05478 – 0.22148	1.19097	175.00000
ml_Methane_per_Hr	-1.35948 *	0.54591	-0.21939	0.08810	-2.43689 – -0.28208	-0.39326 – -0.04552	-2.49032	175.00000
sCOD_Removal	-724.75896	1082.43937	-0.05154	0.07698	-2861.07479 – 1411.55687	-0.20347 – 0.10038	-0.66956	175.00000
pCOD_Washout	-0.21650 ***	0.04113	-0.42738	0.08120	-0.29768 – -0.13532	-0.58763 – -0.26713	-5.26347	175.00000
Observations	180							
R^2 / R^2 adjusted	0.143 / 0.123							

* p<0.05   ** p<0.01   *** p<0.001

	M5-Richness							
Predictors	*Estimates*	*std. Error*	*std. Beta*	*standardized std. Error*	*CI*	*standardized CI*	*Statistic*	*df*
(Intercept)	2088.23394	1160.42708	0.00000	0.06992	-202.09105 – 4378.55893	-0.13800 – 0.13800	0.00000	174.00000
Status_XS	17.24580	28.65653	0.04358	0.07241	-39.31334 – 73.80494	-0.09934 – 0.18650	0.60181	174.00000
Status_S	37.29651	28.65653	0.09425	0.07241	-19.26263 – 93.85566	-0.04868 – 0.23717	1.30150	174.00000
ml_Methane_per_Hr	-1.35948 *	0.54690	-0.21939	0.08826	-2.43891 – -0.28006	-0.39358 – -0.04520	-2.48578	174.00000
sCOD_Removal	-724.75896	1084.41737	-0.05154	0.07712	-2865.06423 – 1415.54632	-0.20375 – 0.10067	-0.66834	174.00000
pCOD_Washout	-0.21650 ***	0.04121	-0.42738	0.08135	-0.29783 – -0.13517	-0.58793 – -0.26683	-5.25387	174.00000
Observations	180							
R^2 / R^2 adjusted	0.145 / 0.120							

* $p<0.05$ ** $p<0.01$ *** $p<0.001$

	M6-Richness							
Predictors	*Estimates*	*std. Error*	*std. Beta*	*standardized std. Error*	*CI*	*standardized CI*	*Statistic*	*df*
(Intercept)	2085.10249	1163.50092	0.00000	0.07010	-211.38227 – 4381.58725	-0.13837 – 0.13837	0.00000	173.00000
Status_XS	20.37725	30.47419	0.05149	0.07701	-39.77183 – 80.52633	-0.10050 – 0.20349	0.66867	173.00000
Status_S	40.42796	30.47419	0.10216	0.07701	-19.72112 – 100.57704	-0.04984 – 0.25416	1.32663	173.00000
Status_XL	9.39434	30.47419	0.02374	0.07701	-50.75474 – 69.54342	-0.12826 – 0.17574	0.30827	173.00000
ml_Methane_per_Hr	-1.35948 *	0.54833	-0.21939	0.08849	-2.44177 – -0.27720	-0.39404 – -0.04473	-2.47931	173.00000
sCOD_Removal	-724.75896	1087.24843	-0.05154	0.07732	-2870.73874 – 1421.22082	-0.20415 – 0.10107	-0.66660	173.00000
pCOD_Washout	-0.21650 ***	0.04132	-0.42738	0.08156	-0.29805 – -0.13495	-0.58836 – -0.26640	-5.24019	173.00000
Observations	180							
R^2 / R^2 adjusted	0.145 / 0.115							

* p<0.05   ** p<0.01   *** p<0.001

	M7-Richness							
Predictors	*Estimates*	*std. Error*	*std. Beta*	*standardized std. Error*	*CI*	*standardized CI*	*Statistic*	*df*
(Intercept)	2094.49683	1166.95942	0.00000	0.07030	-208.90855 – 4397.90221	-0.13877 – 0.13877	0.00000	172.00000
Status_XS	10.98291	35.28913	0.02775	0.08918	-58.67261 – 80.63843	-0.14826 – 0.20377	0.31123	172.00000
Status_S	31.03362	35.28913	0.07842	0.08918	-38.62189 – 100.68914	-0.09760 – 0.25444	0.87941	172.00000
Status_M	-11.58469	35.28913	-0.02927	0.08918	-81.24021 – 58.07082	-0.20529 – 0.14674	-0.32828	172.00000
Status_L	-7.20398	35.28913	-0.01820	0.08918	-76.85950 – 62.45154	-0.19422 – 0.15781	-0.20414	172.00000
ml_Methane_per_Hr	-1.35948 *	0.54990	-0.21939	0.08874	-2.44490 – -0.27406	-0.39455 – -0.04423	-2.47224	172.00000
sCOD_Removal	-724.75896	1090.35561	-0.05154	0.07754	-2876.95974 – 1427.44182	-0.20460 – 0.10151	-0.66470	172.00000
pCOD_Washout	-0.21650 ***	0.04143	-0.42738	0.08179	-0.29828 – -0.13472	-0.58883 – -0.26594	-5.22526	172.00000
Observations	180							
R^2 / R^2 adjusted	0.145 / 0.110							

** p<0.05   ** p<0.01   *** p<0.001*

Subset Regression Analysis – Shannon Diversity

Model Number	Model	Cross-validation Errors
M4	Shannon ~ Status_XS + Status_S + ml_Methane_per_Hr + pCOD_Washout	0.20644
M5	Shannon ~ Status_XS + Status_S + Status_M + ml_Methane_per_Hr + pCOD_Washout	0.20650
M3	Shannon ~ Status_S + ml_Methane_per_Hr + pCOD_Washout	0.20692
M6	Shannon ~ Status_XS + Status_S + Status_M + Status_L + ml_Methane_per_Hr + pCOD_Washout	0.20758
M2	Shannon ~ ml_Methane_per_Hr + pCOD_Washout	0.21091
M7	Shannon ~ Status_XS + Status_S + Status_M + Status_L + ml_Methane_per_Hr + sCOD_Removal + pCOD_Washout	0.21172
M1	Shannon ~ pCOD_Washout	0.22149

	M1-Shannon							
Predictors	*Estimates*	*std. Error*	*std. Beta*	*standardized std. Error*	*CI*	*standardized CI*	*Statistic*	*df*
(Intercept)	5.33540 ***	0.08124	0.00000	0.07006	5.17508 – 5.49573	-0.13826 – 0.13826	0.00000	178.00000
pCOD_Washout	-0.00026 ***	0.00005	-0.34835	0.07026	-0.00037 – -0.00016	-0.48699 – -0.20970	-4.95809	178.00000
Observations	180							
R^2 / R^2 adjusted	0.121 / 0.116							

* *p<0.05* ** *p<0.01* *** *p<0.001*

	M2-Shannon							
Predictors	*Estimates*	*std. Error*	*std. Beta*	*standardized std. Error*	*CI*	*standardized CI*	*Statistic*	*df*
(Intercept)	6.58874 ***	0.28680	0.00000	0.06650	6.02276 – 7.15472	-0.13123 – 0.13123	0.00000	177.00000
ml_Methane_per_Hr	-0.00321 ***	0.00071	-0.34731	0.07655	-0.00460 – -0.00181	-0.49837 – -0.19624	-4.53718	177.00000
pCOD_Washout	-0.00039 ***	0.00006	-0.51887	0.07655	-0.00051 – -0.00028	-0.66993 – -0.36780	-6.77844	177.00000
Observations	180							
R^2 / R^2 adjusted	0.213 / 0.204							

** p<0.05   ** p<0.01   *** p<0.001*

	**M3-Shannon**							
Predictors	*Estimates*	*std. Error*	*std. Beta*	*standardized std. Error*	*CI*	*standardized CI*	*Statistic*	*df*
(Intercept)	6.56506 ***	0.28025	0.00000	0.06496	6.01198 – 7.11814	-0.12820 – 0.12820	0.00000	176.00000
Status_S	0.11839 **	0.03840	0.20084	0.06514	0.04261 – 0.19416	0.07229 – 0.32939	3.08326	176.00000
ml_Methane_per_Hr	-0.00321 ***	0.00069	-0.34731	0.07477	-0.00457 – -0.00184	-0.49487 – -0.19974	-4.64493	176.00000
pCOD_Washout	-0.00039 ***	0.00006	-0.51887	0.07477	-0.00050 – -0.00028	-0.66643 – -0.37130	-6.93941	176.00000
Observations	180							
R^2 / R^2 adjusted	0.253 / 0.240							

* *p*<0.05   ** *p*<0.01   *** *p*<0.001

	M4-Shannon							
Predictors	*Estimates*	*std. Error*	*std. Beta*	*standardized std. Error*	*CI*	*standardized CI*	*Statistic*	*df*
(Intercept)	6.57875 ***	0.27970	0.00000	0.06479	6.02673 – 7.13076	-0.12787 – 0.12787	0.00000	175.00000
Status_XS	-0.05475	0.03955	-0.09288	0.06710	-0.13281 – 0.02331	-0.22531 – 0.03955	-1.38415	175.00000
Status_S	0.10470 **	0.03955	0.17762	0.06710	0.02664 – 0.18276	0.04519 – 0.31005	2.64707	175.00000
ml_Methane_per_Hr	-0.00321 ***	0.00069	-0.34731	0.07458	-0.00456 – -0.00185	-0.49449 – -0.20012	-4.65700	175.00000
pCOD_Washout	-0.00039 ***	0.00006	-0.51887	0.07458	-0.00050 – -0.00028	-0.66605 – -0.37168	-6.95744	175.00000
Observations	180							
R^2 / R^2 adjusted	0.261 / 0.244							

* $p<0.05$ ** $p<0.01$ *** $p<0.001$

	M5-Shannon							
Predictors	*Estimates*	*std. Error*	*std. Beta*	*standardized std. Error*	*CI*	*standardized CI*	*Statistic*	*df*
(Intercept)	6.57158 ***	0.28064	0.00000	0.06493	6.01768 – 7.12548	-0.12814 – 0.12814	0.00000	174.00000
Status_XS	-0.04758	0.04204	-0.08072	0.07132	-0.13056 – 0.03540	-0.22149 – 0.06005	-1.13176	174.00000
Status_S	0.11187 **	0.04204	0.18978	0.07132	0.02889 – 0.19484	0.04901 – 0.33054	2.66088	174.00000
Status_M	0.02150	0.04204	0.03648	0.07132	-0.06148 – 0.10448	-0.10429 – 0.17724	0.51143	174.00000
ml_Methane_per_Hr	-0.00321 ***	0.00069	-0.34731	0.07473	-0.00457 – -0.00184	-0.49481 – -0.19980	-4.64716	174.00000
pCOD_Washout	-0.00039 ***	0.00006	-0.51887	0.07473	-0.00050 – -0.00028	-0.66637 – -0.37136	-6.94275	174.00000
Observations	180							
R^2 / R^2 adjusted	0.262 / 0.241							

** p<0.05   ** p<0.01   *** p<0.001*

	**M6-Shannon**							
Predictors	*Estimates*	*std. Error*	*std. Beta*	*standardized std. Error*	*CI*	*standardized CI*	*Statistic*	*df*
(Intercept)	6.57754 ***	0.28245	0.00000	0.06510	6.02005 – 7.13504	-0.12850 – 0.12850	0.00000	173.00000
Status_XS	-0.05354	0.04868	-0.09083	0.08258	-0.14962 – 0.04253	-0.25382 – 0.07216	-1.09996	173.00000
Status_S	0.10590 *	0.04868	0.17966	0.08258	0.00983 – 0.20198	0.01667 – 0.34265	2.17568	173.00000
Status_M	0.01554	0.04868	0.02636	0.08258	-0.08054 – 0.11162	-0.13663 – 0.18935	0.31923	173.00000
Status_L	-0.01192	0.04868	-0.02023	0.08258	-0.10800 – 0.08415	-0.18322 – 0.14276	-0.24496	173.00000
ml_Methane_per_Hr	-0.00321 ***	0.00069	-0.34731	0.07494	-0.00457 – -0.00184	-0.49522 – -0.19940	-4.63460	173.00000
pCOD_Washout	-0.00039 ***	0.00006	-0.51887	0.07494	-0.00050 – -0.00028	-0.66678 – -0.37096	-6.92397	173.00000
Observations	180							
R^2 / R^2 adjusted	0.263 / 0.237							

* p<0.05   ** p<0.01   *** p<0.001

	M7-Shannon							
Predictors	*Estimates*	*std. Error*	*std. Beta*	*standardized std. Error*	*CI*	*standardized CI*	*Statistic*	*df*
(Intercept)	6.43753 ***	1.61430	0.00000	0.06529	3.25114 – 9.62392	-0.12887 – 0.12887	0.00000	172.00000
Status_XS	-0.05354	0.04882	-0.09083	0.08282	-0.14990 – 0.04281	-0.25430 – 0.07263	-1.09681	172.00000
Status_S	0.10590 *	0.04882	0.17966	0.08282	0.00955 – 0.20226	0.01620 – 0.34313	2.16943	172.00000
Status_M	0.01554	0.04882	0.02636	0.08282	-0.08082 – 0.11190	-0.13711 – 0.18983	0.31831	172.00000
Status_L	-0.01192	0.04882	-0.02023	0.08282	-0.10828 – 0.08443	-0.18370 – 0.14324	-0.24426	172.00000
ml_Methane_per_Hr	-0.00318 ***	0.00076	-0.34433	0.08241	-0.00468 – -0.00168	-0.50700 – -0.18166	-4.17807	172.00000
sCOD_Removal	0.13288	1.50833	0.00634	0.07201	-2.84434 – 3.11010	-0.13580 – 0.14848	0.08810	172.00000
pCOD_Washout	-0.00039 ***	0.00006	-0.51789	0.07596	-0.00050 – -0.00028	-0.66783 – -0.36796	-6.81809	172.00000
Observations	180							
R^2 / R^2 adjusted	0.263 / 0.233							

** p<0.05   ** p<0.01   *** p<0.001*

Subset Regression Analysis – Local Contribution to Beta Diversity (Bray Curtis)

Model Number	Model	Cross-validation Errors
M4	LCBD ~ Status_XS + Status_S + Status_XL + sCOD_Removal	0.00084
M3	LCBD ~ Status_XS + Status_XL + sCOD_Removal	0.00084
M2	LCBD ~ Status_XS + sCOD_Removal	0.00084
M1	LCBD ~ Status_XS	0.00085
M6	LCBD ~ Status_XS + Status_S + Status_XL + ml_Methane_per_Hr + sCOD_Removal + pCOD_Washout	0.00085
M7	LCBD ~ Status_XS + Status_S + Status_M + Status_L + ml_Methane_per_Hr + sCOD_Removal + pCOD_Washout	0.00085
M5	LCBD ~ Status_XS + Status_XL + ml_Methane_per_Hr + sCOD_Removal + pCOD_Washout	0.00085

	M1-LCBD (Bray)							
Predictors	*Estimates*	*std. Error*	*std. Beta*	*standardized std. Error*	*CI*	*standardized CI*	*Statistic*	*df*
(Intercept)	0.00489 ***	0.00007	0.00000	0.04007	0.00475 – 0.00503	-0.07906 – 0.07906	0.00000	178.00000
Status_XS	0.00332 ***	0.00016	0.84420	0.04018	0.00301 – 0.00364	0.76492 – 0.92349	21.01217	178.00000
Observations	180							
R^2 / R^2 adjusted	0.713 / 0.711							

* *p<0.05* ** *p<0.01* *** *p<0.001*

	M2- LCBD (Bray)							
Predictors	*Estimates*	*std. Error*	*std. Beta*	*standardized std. Error*	*CI*	*standardized CI*	*Statistic*	*df*
(Intercept)	0.02056 ***	0.00538	0.00000	0.03925	0.00995 – 0.03118	-0.07745 – 0.07745	0.00000	177.00000
Status_XS	0.00332 ***	0.00015	0.84420	0.03936	0.00302 – 0.00363	0.76653 – 0.92187	21.44958	177.00000
sCOD_Removal	-0.01604 **	0.00551	-0.11467	0.03936	-0.02691 – -0.00518	-0.19234 – -0.03699	-2.91343	177.00000
Observations	180							
R^2 / R^2 adjusted	0.726 / 0.723							

* *p<0.05* ** *p<0.01* *** *p<0.001*

	**M3- LCBD (Bray)**							
Predictors	*Estimates*	*std. Error*	*std. Beta*	*standardized std. Error*	*CI*	*standardized CI*	*Statistic*	*df*
(Intercept)	0.02051 ***	0.00537	0.00000	0.03915	0.00992 – 0.03110	-0.07726 – 0.07726	0.00000	176.00000
Status_XS	0.00338 ***	0.00016	0.85811	0.04055	0.00306 – 0.00369	0.77809 – 0.93814	21.16336	176.00000
Status_XL	0.00022	0.00016	0.05565	0.04055	-0.00010 – 0.00053	-0.02437 – 0.13567	1.37245	176.00000
sCOD_Removal	-0.01604 **	0.00549	-0.11467	0.03926	-0.02688 – -0.00520	-0.19215 – -0.03718	-2.92069	176.00000
Observations	180							
R^2 / R^2 adjusted	0.729 / 0.724							

* *p<0.05* ** *p<0.01* *** *p<0.001*

	M4- LCBD (Bray)							
Predictors	*Estimates*	*std. Error*	*std. Beta*	*standardized std. Error*	*CI*	*standardized CI*	*Statistic*	*df*
(Intercept)	0.02044 ***	0.00536	0.00000	0.03910	0.00986 – 0.03102	-0.07718 – 0.07718	0.00000	175.00000
Status_XS	0.00345 ***	0.00017	0.87518	0.04296	0.00311 – 0.00378	0.79040 – 0.95996	20.37407	175.00000
Status_S	0.00020	0.00017	0.05119	0.04296	-0.00013 – 0.00054	-0.03358 – 0.13597	1.19176	175.00000
Status_XL	0.00029	0.00017	0.07271	0.04296	-0.00005 – 0.00062	-0.01206 – 0.15749	1.69276	175.00000
sCOD_Removal	-0.01604 **	0.00549	-0.11467	0.03921	-0.02687 – -0.00521	-0.19206 – -0.03727	-2.92417	175.00000
Observations	180							
R^2 / R^2 adjusted	0.731 / 0.725							

* *p*<0.05   ** *p*<0.01   *** *p*<0.001

	M5- LCBD (Bray)							
Predictors	*Estimates*	*std. Error*	*std. Beta*	*standardized std. Error*	*CI*	*standardized CI*	*Statistic*	*df*
(Intercept)	0.01504 *	0.00645	0.00000	0.03907	0.00231 – 0.02777	-0.07712 – 0.07712	0.00000	174.00000
Status_XS	0.00338 ***	0.00016	0.85811	0.04047	0.00306 – 0.00369	0.77825 – 0.93798	21.20576	174.00000
Status_XL	0.00022	0.00016	0.05565	0.04047	-0.00010 – 0.00053	-0.02422 – 0.13552	1.37520	174.00000
ml_Methane_per_Hr	0.00000	0.00000	0.07595	0.04932	-0.00000 – 0.00001	-0.02139 – 0.17329	1.54001	174.00000
sCOD_Removal	-0.01248 *	0.00603	-0.08919	0.04309	-0.02438 – -0.00058	-0.17424 – -0.00413	-2.06958	174.00000
pCOD_Washout	0.00000	0.00000	0.05790	0.04546	-0.00000 – 0.00000	-0.03181 – 0.14762	1.27383	174.00000
Observations	180							
R^2 / R^2 adjusted	0.733 / 0.725							

** p<0.05   ** p<0.01   *** p<0.001*

	M6- LCBD (Bray)							
Predictors	*Estimates*	*std. Error*	*std. Beta*	*standardized std. Error*	*CI*	*standardized CI*	*Statistic*	*df*
(Intercept)	0.01497 *	0.00644	0.00000	0.03902	0.00225 – 0.02769	-0.07703 – 0.07703	0.00000	173.00000
Status_XS	0.00345 ***	0.00017	0.87518	0.04287	0.00311 – 0.00378	0.79057 – 0.95979	20.41550	173.00000
Status_S	0.00020	0.00017	0.05119	0.04287	-0.00013 – 0.00053	-0.03342 – 0.13581	1.19419	173.00000
Status_XL	0.00029	0.00017	0.07271	0.04287	-0.00005 – 0.00062	-0.01190 – 0.15733	1.69620	173.00000
ml_Methane_per_Hr	0.00000	0.00000	0.07595	0.04926	-0.00000 – 0.00001	-0.02127 – 0.17318	1.54189	173.00000
sCOD_Removal	-0.01248 *	0.00602	-0.08919	0.04304	-0.02436 – -0.00059	-0.17414 – -0.00423	-2.07211	173.00000
pCOD_Washout	0.00000	0.00000	0.05790	0.04540	-0.00000 – 0.00000	-0.03171 – 0.14752	1.27539	173.00000
Observations	180							
R^2 / R^2 adjusted	0.735 / 0.726							

* *p<0.05   ** p<0.01   *** p<0.001*

	M7- LCBD (Bray)							
Predictors	*Estimates*	*std. Error*	*std. Beta*	*standardized std. Error*	*CI*	*standardized CI*	*Statistic*	*df*
(Intercept)	0.01526 *	0.00646	0.00000	0.03913	0.00250 – 0.02801	-0.07723 – 0.07723	0.00000	172.00000
Status_XS	0.00316 ***	0.00020	0.80247	0.04963	0.00277 – 0.00354	0.70451 – 0.90042	16.16960	172.00000
Status_S	-0.00008	0.00020	-0.02152	0.04963	-0.00047 – 0.00030	-0.11948 – 0.07644	-0.43363	172.00000
Status_M	-0.00032	0.00020	-0.08091	0.04963	-0.00070 – 0.00007	-0.17887 – 0.01705	-1.63033	172.00000
Status_L	-0.00025	0.00020	-0.06452	0.04963	-0.00064 – 0.00013	-0.16247 – 0.03344	-1.30000	172.00000
ml_Methane_per_Hr	0.00000	0.00000	0.07595	0.04939	-0.00000 – 0.00001	-0.02153 – 0.17343	1.53792	172.00000
sCOD_Removal	-0.01248 *	0.00604	-0.08919	0.04315	-0.02439 – -0.00056	-0.17437 – -0.00401	-2.06677	172.00000
pCOD_Washout	0.00000	0.00000	0.05790	0.04552	-0.00000 – 0.00000	-0.03194 – 0.14775	1.27210	172.00000
Observations	180							
R^2 / R^2 adjusted	0.735 / 0.724							

** p<0.05   ** p<0.01   *** p<0.001*

Subset Regression Analysis – Local Contribution to Beta Diversity (Unifrac)

Model Number	Model	Cross-validation Errors
M3	LCBD ~ Status_XS + Status_XL + sCOD_Removal	0.00048
M4	LCBD ~ Status_XS + Status_XL + ml_Methane_per_Hr + sCOD_Removal	0.00048
M2	LCBD ~ Status_XS + sCOD_Removal	0.00048
M5	LCBD ~ Status_XS + Status_XL + ml_Methane_per_Hr + sCOD_Removal + pCOD_Washout	0.00049
M6	LCBD ~ Status_XS + Status_M + Status_XL + ml_Methane_per_Hr + sCOD_Removal + pCOD_Washout	0.00049
M1	LCBD ~ Status_XS	0.00049
M7	LCBD ~ Status_XS + Status_S + Status_M + Status_L + ml_Methane_per_Hr + sCOD_Removal + pCOD_Washout	0.00049

	M1-LCBD (Unifrac)							
Predictors	*Estimates*	*std. Error*	*std. Beta*	*standardized std. Error*	*CI*	*standardized CI*	*Statistic*	*df*
(Intercept)	0.00542 ***	0.00004	0.00000	0.06555	0.00534 – 0.00550	-0.12936 – 0.12936	0.00000	178.00000
Status_XS	0.00068 ***	0.00009	0.48045	0.06574	0.00050 – 0.00086	0.35073 – 0.61017	7.30886	178.00000
Observations	180							
R^2 / R^2 adjusted	0.231 / 0.227							

* *p<0.05* ** *p<0.01* *** *p<0.001*

	M2-LCBD (Unifrac)							
Predictors	*Estimates*	*std. Error*	*std. Beta*	*standardized std. Error*	*CI*	*standardized CI*	*Statistic*	*df*
(Intercept)	0.01502 ***	0.00315	0.00000	0.06408	0.00880 – 0.02125	-0.12646 – 0.12646	0.00000	177.00000
Status_XS	0.00068 ***	0.00009	0.48045	0.06426	0.00050 – 0.00086	0.35364 – 0.60726	7.47697	177.00000
sCOD_Removal	-0.00983 **	0.00323	-0.19577	0.06426	-0.01620 – -0.00346	-0.32258 – -0.06896	-3.04667	177.00000
Observations	180							
R^2 / R^2 adjusted	0.269 / 0.261							

* *p<0.05*   ** *p<0.01*   *** *p<0.001*

	M3-LCBD (Unifrac)							
Predictors	*Estimates*	*std. Error*	*std. Beta*	*standardized std. Error*	*CI*	*standardized CI*	*Statistic*	*df*
(Intercept)	0.01498 ***	0.00312	0.00000	0.06351	0.00881 – 0.02114	-0.12534 – 0.12534	0.00000	176.00000
Status_XS	0.00073 ***	0.00009	0.51414	0.06577	0.00054 – 0.00091	0.38433 – 0.64394	7.81678	176.00000
Status_XL	0.00019 *	0.00009	0.13475	0.06577	0.00001 – 0.00037	0.00494 – 0.26455	2.04863	176.00000
sCOD_Removal	-0.00983 **	0.00320	-0.19577	0.06369	-0.01614 – -0.00352	-0.32146 – -0.07009	-3.07406	176.00000
Observations	180							
R^2 / R^2 adjusted	0.286 / 0.274							

* *p*<0.05   ** *p*<0.01   *** *p*<0.001

	**M4-LCBD (Unifrac)**							
Predictors	*Estimates*	*std. Error*	*std. Beta*	*standardized std. Error*	*CI*	*standardized CI*	*Statistic*	*df*
(Intercept)	0.01245 ***	0.00362	0.00000	0.06335	0.00531 – 0.01960	-0.12503 – 0.12503	0.00000	175.00000
Status_XS	0.00073 ***	0.00009	0.51414	0.06561	0.00054 – 0.00091	0.38465 – 0.64363	7.83617	175.00000
Status_XL	0.00019 *	0.00009	0.13475	0.06561	0.00001 – 0.00037	0.00526 – 0.26424	2.05371	175.00000
ml_Methane_per_Hr	0.00000	0.00000	0.09464	0.06913	-0.00000 – 0.00001	-0.04180 – 0.23108	1.36894	175.00000
sCOD_Removal	-0.00796 *	0.00347	-0.15844	0.06913	-0.01481 – -0.00110	-0.29488 – -0.02200	-2.29188	175.00000
Observations	180							
R^2 / R^2 adjusted	0.294 / 0.278							

* *p*<0.05   ** *p*<0.01   *** *p*<0.001

	M5-LCBD (Unifrac)							
Predictors	*Estimates*	*std. Error*	*std. Beta*	*standardized std. Error*	*CI*	*standardized CI*	*Statistic*	*df*
(Intercept)	0.01149 **	0.00376	0.00000	0.06336	0.00408 – 0.01890	-0.12506 – 0.12506	0.00000	174.00000
Status_XS	0.00073 ***	0.00009	0.51414	0.06562	0.00054 – 0.00091	0.38462 – 0.64366	7.83481	174.00000
Status_XL	0.00019 *	0.00009	0.13475	0.06562	0.00001 – 0.00037	0.00523 – 0.26426	2.05335	174.00000
ml_Methane_per_Hr	0.00000	0.00000	0.13360	0.07998	-0.00000 – 0.00001	-0.02426 – 0.29145	1.67042	174.00000
sCOD_Removal	-0.00746 *	0.00351	-0.14861	0.06989	-0.01439 – -0.00054	-0.28654 – -0.01067	-2.12641	174.00000
pCOD_Washout	0.00000	0.00000	0.07145	0.07372	-0.00000 – 0.00000	-0.07404 – 0.21694	0.96927	174.00000
Observations	180							
R^2 / R^2 adjusted	0.298 / 0.277							

* p<0.05   ** p<0.01   *** p<0.001

	M6-LCBD (Unifrac)							
Predictors	*Estimates*	*std. Error*	*std. Beta*	*standardized std. Error*	*CI*	*standardized CI*	*Statistic*	*df*
(Intercept)	0.01152 **	0.00376	0.00000	0.06339	0.00410 – 0.01894	-0.12511 – 0.12511	0.00000	173.00000
Status_XS	0.00070 ***	0.00010	0.49271	0.06963	0.00050 – 0.00089	0.35527 – 0.63015	7.07588	173.00000
Status_M	-0.00009	0.00010	-0.06428	0.06963	-0.00029 – 0.00010	-0.20172 – 0.07316	-0.92313	173.00000
Status_XL	0.00016	0.00010	0.11332	0.06963	-0.00003 – 0.00035	-0.02412 – 0.25076	1.62739	173.00000
ml_Methane_per_Hr	0.00000	0.00000	0.13360	0.08001	-0.00000 – 0.00001	-0.02433 – 0.29153	1.66971	173.00000
sCOD_Removal	-0.00746 *	0.00351	-0.14861	0.06992	-0.01439 – -0.00053	-0.28660 – -0.01061	-2.12551	173.00000
pCOD_Washout	0.00000	0.00000	0.07145	0.07375	-0.00000 – 0.00000	-0.07411 – 0.21701	0.96886	173.00000
Observations	180							
R^2 / R^2 adjusted	0.301 / 0.277							

* p<0.05   ** p<0.01   *** p<0.001

	M7-LCBD (Unifrac)							
Predictors	*Estimates*	*std. Error*	*std. Beta*	*standardized std. Error*	*CI*	*standardized CI*	*Statistic*	*df*
(Intercept)	0.01168 **	0.00377	0.00000	0.06357	0.00424 – 0.01912	-0.12547 – 0.12547	0.00000	172.00000
Status_XS	0.00054 ***	0.00011	0.37939	0.08063	0.00031 – 0.00076	0.22024 – 0.53855	4.70524	172.00000
Status_S	-0.00015	0.00011	-0.10676	0.08063	-0.00038 – 0.00007	-0.26591 – 0.05240	-1.32399	172.00000
Status_M	-0.00025 *	0.00011	-0.17760	0.08063	-0.00048 – -0.00003	-0.33675 – -0.01844	-2.20259	172.00000
Status_L	-0.00017	0.00011	-0.11988	0.08063	-0.00039 – 0.00006	-0.27904 – 0.03927	-1.48678	172.00000
ml_Methane_per_Hr	0.00000	0.00000	0.13360	0.08024	-0.00000 – 0.00001	-0.02478 – 0.29198	1.66501	172.00000
sCOD_Removal	-0.00746 *	0.00352	-0.14861	0.07011	-0.01441 – -0.00051	-0.28700 – -0.01021	-2.11952	172.00000
pCOD_Washout	0.00000	0.00000	0.07145	0.07396	-0.00000 – 0.00000	-0.07453 – 0.21743	0.96613	172.00000
Observations	180							
R^2 / R^2 adjusted	0.301 / 0.273							

* $p<0.05$ ** $p<0.01$ *** $p<0.001$

Subset Regression Analysis – Local Contribution to Beta Diversity (Weighted Unifrac)

Model Number	Model	Cross-validation Errors
M4	LCBD ~ Status_XS + ml_Methane_per_Hr + sCOD_Removal + pCOD_Washout	0.00146
M3	LCBD ~ Status_XS + ml_Methane_per_Hr + pCOD_Washout	0.00146
M5	LCBD ~ Status_XS + Status_M + ml_Methane_per_Hr + sCOD_Removal + pCOD_Washout	0.00146
M2	LCBD ~ Status_XS + pCOD_Washout	0.00146
M6	LCBD ~ Status_XS + Status_M + Status_XL + ml_Methane_per_Hr + sCOD_Removal + pCOD_Washout	0.00147
M1	LCBD ~ Status_XS	0.00147
M7	LCBD ~ Status_XS + Status_S + Status_M + Status_L + ml_Methane_per_Hr + sCOD_Removal + pCOD_Washout	0.00148

	M1-LCBD (Wunifrac)							
Predictors	*Estimates*	*std. Error*	*std. Beta*	*standardized std. Error*	*CI*	*standardized CI*	*Statistic*	*df*
(Intercept)	0.00513 ***	0.00012	-0.00000	0.06480	0.00489 – 0.00538	-0.12788 – 0.12788	-0.00000	178.00000
Status_XS	0.00211 ***	0.00028	0.49837	0.06498	0.00157 – 0.00266	0.37014 – 0.62661	7.66947	178.00000
Observations	180							
R^2 / R^2 adjusted	0.248 / 0.244							

* *p<0.05* ** *p<0.01* *** *p<0.001*

	M2-LCBD (Wunifrac)							
Predictors	*Estimates*	*std. Error*	*std. Beta*	*standardized std. Error*	*CI*	*standardized CI*	*Statistic*	*df*
(Intercept)	0.00654 ***	0.00053	-0.00000	0.06369	0.00548 – 0.00759	-0.12568 – 0.12568	-0.00000	177.00000
Status_XS	0.00211 ***	0.00027	0.49837	0.06386	0.00158 – 0.00265	0.37234 – 0.62441	7.80374	177.00000
pCOD_Washout	-0.00000 **	0.00000	-0.17240	0.06386	-0.00000 – -0.00000	-0.29843 – -0.04637	-2.69947	177.00000
Observations	180							
R^2 / R^2 adjusted	0.278 / 0.270							

* *p<0.05   ** p<0.01   *** p<0.001*

	M3-LCBD (Wunifrac)							
Predictors	*Estimates*	*std. Error*	*std. Beta*	*standardized std. Error*	*CI*	*standardized CI*	*Statistic*	*df*
(Intercept)	0.01118 ***	0.00195	-0.00000	0.06278	0.00733 – 0.01502	-0.12391 – 0.12391	-0.00000	176.00000
Status_XS	0.00211 ***	0.00027	0.49837	0.06296	0.00159 – 0.00264	0.37412 – 0.62263	7.91590	176.00000
ml_Methane_per_Hr	-0.00001 *	0.00000	-0.17884	0.07227	-0.00002 – -0.00000	-0.32147 – -0.03622	-2.47470	176.00000
pCOD_Washout	-0.00000 ***	0.00000	-0.26020	0.07227	-0.00000 – -0.00000	-0.40283 – -0.11758	-3.60052	176.00000
Observations	180							
R^2 / R^2 adjusted	0.302 / 0.290							

* *p<0.05*   ** *p<0.01*   *** *p<0.001*

	M4-LCBD (Wunifrac)							
Predictors	*Estimates*	*std. Error*	*std. Beta*	*standardized std. Error*	*CI*	*standardized CI*	*Statistic*	*df*
(Intercept)	0.03575 **	0.01103	0.00000	0.06206	0.01398 – 0.05753	-0.12249 – 0.12249	0.00000	175.00000
Status_XS	0.00211 ***	0.00026	0.49837	0.06223	0.00159 – 0.00263	0.37555 – 0.62120	8.00798	175.00000
ml_Methane_per_Hr	-0.00002 **	0.00001	-0.25158	0.07834	-0.00003 – -0.00001	-0.40618 – -0.09697	-3.21141	175.00000
sCOD_Removal	-0.02333 *	0.01031	-0.15487	0.06845	-0.04367 – -0.00298	-0.28996 – -0.01977	-2.26242	175.00000
pCOD_Washout	-0.00000 ***	0.00000	-0.28393	0.07220	-0.00000 – -0.00000	-0.42643 – -0.14143	-3.93236	175.00000
Observations	180							
R^2 / R^2 adjusted	0.322 / 0.307							

* p<0.05   ** p<0.01   *** p<0.001

	M5-LCBD (Wunifrac)							
Predictors	*Estimates*	*std. Error*	*std. Beta*	*standardized std. Error*	*CI*	*standardized CI*	*Statistic*	*df*
(Intercept)	0.03568 **	0.01103	0.00000	0.06203	0.01392 – 0.05745	-0.12243 – 0.12243	0.00000	174.00000
Status_XS	0.00219 ***	0.00027	0.51582	0.06424	0.00165 – 0.00272	0.38902 – 0.64261	8.02918	174.00000
Status_M	0.00030	0.00027	0.06977	0.06424	-0.00024 – 0.00083	-0.05702 – 0.19657	1.08605	174.00000
ml_Methane_per_Hr	-0.00002 **	0.00001	-0.25158	0.07830	-0.00003 – -0.00001	-0.40611 – -0.09704	-3.21306	174.00000
sCOD_Removal	-0.02333 *	0.01030	-0.15487	0.06842	-0.04366 – -0.00299	-0.28990 – -0.01983	-2.26358	174.00000
pCOD_Washout	-0.00000 ***	0.00000	-0.28393	0.07217	-0.00000 – -0.00000	-0.42636 – -0.14150	-3.93438	174.00000
Observations	180							
R^2 / R^2 adjusted	0.327 / 0.307							

* p<0.05   ** p<0.01   *** p<0.001

	M6-LCBD (Wunifrac)							
Predictors	*Estimates*	*std. Error*	*std. Beta*	*standardized std. Error*	*CI*	*standardized CI*	*Statistic*	*df*
(Intercept)	0.03573 **	0.01105	0.00000	0.06217	0.01391 – 0.05754	-0.12270 – 0.12270	0.00000	173.00000
Status_XS	0.00214 ***	0.00029	0.50462	0.06829	0.00157 – 0.00271	0.36983 – 0.63940	7.38949	173.00000
Status_M	0.00025	0.00029	0.05857	0.06829	-0.00032 – 0.00082	-0.07621 – 0.19336	0.85771	173.00000
Status_XL	-0.00014	0.00029	-0.03360	0.06829	-0.00071 – 0.00043	-0.16838 – 0.10119	-0.49197	173.00000
ml_Methane_per_Hr	-0.00002 **	0.00001	-0.25158	0.07847	-0.00003 – -0.00001	-0.40646 – -0.09670	-3.20606	173.00000
sCOD_Removal	-0.02333 *	0.01033	-0.15487	0.06857	-0.04371 – -0.00294	-0.29020 – -0.01953	-2.25865	173.00000
pCOD_Washout	-0.00000 ***	0.00000	-0.28393	0.07232	-0.00000 – -0.00000	-0.42668 – -0.14118	-3.92580	173.00000
Observations	180							
R^2 / R^2 adjusted	0.328 / 0.304							

* $p<0.05$ ** $p<0.01$ *** $p<0.001$

	M7-LCBD (Wunifrac)							
Predictors	*Estimates*	*std. Error*	*std. Beta*	*standardized std. Error*	*CI*	*standardized CI*	*Statistic*	*df*
(Intercept)	0.03559 **	0.01108	0.00000	0.06234	0.01371 – 0.05746	-0.12304 – 0.12304	0.00000	172.00000
Status_XS	0.00228 ***	0.00034	0.53821	0.07907	0.00162 – 0.00294	0.38214 – 0.69429	6.80678	172.00000
Status_S	0.00010	0.00034	0.02474	0.07907	-0.00056 – 0.00077	-0.13134 – 0.18081	0.31286	172.00000
Status_M	0.00039	0.00034	0.09217	0.07907	-0.00027 – 0.00105	-0.06391 – 0.24824	1.16565	172.00000
Status_L	0.00018	0.00034	0.04245	0.07907	-0.00048 – 0.00084	-0.11362 – 0.19853	0.53692	172.00000
ml_Methane_per_Hr	-0.00002 **	0.00001	-0.25158	0.07869	-0.00003 – -0.00001	-0.40689 – -0.09626	-3.19724	172.00000
sCOD_Removal	-0.02333 *	0.01036	-0.15487	0.06875	-0.04377 – -0.00288	-0.29058 – -0.01915	-2.25244	172.00000
pCOD_Washout	-0.00000 ***	0.00000	-0.28393	0.07252	-0.00000 – -0.00000	-0.42708 – -0.14078	-3.91501	172.00000
Observations	180							
R^2 / R^2 adjusted	0.328 / 0.301							

* $p<0.05$ ** $p<0.01$ *** $p<0.001$

Subset Regression Analysis – Net Relatedness Index (NRI)

Model Number	Model	Cross-validation Errors
M5	NRI ~ Status_XS + Status_XL + ml_Methane_per_Hr + sCOD_Removal + pCOD_Washout	0.85114
M6	NRI ~ Status_XS + Status_S + Status_XL + ml_Methane_per_Hr + sCOD_Removal + pCOD_Washout	0.85343
M7	NRI ~ Status_XS + Status_S + Status_M + Status_L + ml_Methane_per_Hr + sCOD_Removal + pCOD_Washout	0.85642
M4	NRI ~ Status_XS + ml_Methane_per_Hr + sCOD_Removal + pCOD_Washout	0.85674
M3	NRI ~ Status_XS + sCOD_Removal + pCOD_Washout	0.88019
M2	NRI ~ Status_XS + sCOD_Removal	0.88779
M1	NRI ~ Status_XS	0.90429

	M1-NRI							
Predictors	*Estimates*	*std. Error*	*std. Beta*	*standardized std. Error*	*CI*	*standardized CI*	*Statistic*	*df*
(Intercept)	0.95853 ***	0.07573	0.00000	0.07275	0.80909 – 1.10798	-0.14356 – 0.14356	0.00000	178.00000
Status_XS	-0.53298 **	0.16934	-0.22961	0.07295	-0.86715 – -0.19882	-0.37357 – -0.08565	-3.14748	178.00000
Observations	180							
R^2 / R^2 adjusted	0.053 / 0.047							

* *p<0.05*   ** *p<0.01*   *** *p<0.001*

	**M2-NRI**							
Predictors	*Estimates*	*std. Error*	*std. Beta*	*standardized std. Error*	*CI*	*standardized CI*	*Statistic*	*df*
(Intercept)	-14.47142 *	5.78008	-0.00000	0.07153	-25.87816 – -3.06468	-0.14116 – 0.14116	-0.00000	177.00000
Status_XS	-0.53298 **	0.16650	-0.22961	0.07173	-0.86156 – -0.20441	-0.37116 – -0.08806	-3.20120	177.00000
sCOD_Removal	15.79454 **	5.91617	0.19149	0.07173	4.11924 – 27.46984	0.04994 – 0.33304	2.66973	177.00000
Observations	180							
R^2 / R^2 adjusted	0.089 / 0.079							

* p<0.05   ** p<0.01   *** p<0.001

	**M3-NRI**							
Predictors	*Estimates*	*std. Error*	*std. Beta*	*standardized std. Error*	*CI*	*standardized CI*	*Statistic*	*df*
(Intercept)	-14.18870 *	5.71733	-0.00000	0.07073	-25.47204 – -2.90535	-0.13959 – 0.13959	-0.00000	176.00000
Status_XS	-0.53298 **	0.16465	-0.22961	0.07093	-0.85792 – -0.20805	-0.36959 – -0.08963	-3.23712	176.00000
sCOD_Removal	14.77954 *	5.86810	0.17918	0.07114	3.19864 – 26.36044	0.03878 – 0.31959	2.51862	176.00000
pCOD_Washout	0.00047 *	0.00021	0.15901	0.07114	0.00006 – 0.00089	0.01860 – 0.29941	2.23504	176.00000
Observations	180							
R^2 / R^2 adjusted	0.115 / 0.099							

* *p<0.05   ** p<0.01   *** p<0.001*

	M4-NRI							
Predictors	*Estimates*	*std. Error*	*std. Beta*	*standardized std. Error*	*CI*	*standardized CI*	*Statistic*	*df*
(Intercept)	-26.36169 ***	6.70516	-0.00000	0.06888	-39.59509 – -13.12829	-0.13594 – 0.13594	-0.00000	175.00000
Status_XS	-0.53298 **	0.16033	-0.22961	0.06907	-0.84941 – -0.21656	-0.36593 – -0.09329	-3.32434	175.00000
ml_Methane_per_Hr	0.01029 **	0.00316	0.28322	0.08694	0.00406 – 0.01653	0.11163 – 0.45480	3.25755	175.00000
sCOD_Removal	23.15617 ***	6.26609	0.28074	0.07597	10.78934 – 35.52299	0.13081 – 0.43067	3.69548	175.00000
pCOD_Washout	0.00086 ***	0.00024	0.29020	0.08013	0.00039 – 0.00133	0.13205 – 0.44835	3.62150	175.00000
Observations	180							
R^2 / R^2 adjusted	0.165 / 0.146							

* $p<0.05$ ** $p<0.01$ *** $p<0.001$

	M5-NRI							
Predictors	*Estimates*	*std. Error*	*std. Beta*	*standardized std. Error*	*CI*	*standardized CI*	*Statistic*	*df*
(Intercept)	-26.44113 ***	6.65340	-0.00000	0.06834	-39.57289 – -13.30937	-0.13489 – 0.13489	-0.00000	174.00000
Status_XS	-0.45354 **	0.16430	-0.19539	0.07078	-0.77783 – -0.12926	-0.33509 – -0.05568	-2.76039	174.00000
Status_XL	0.31776	0.16430	0.13689	0.07078	-0.00653 – 0.64205	-0.00281 – 0.27660	1.93398	174.00000
ml_Methane_per_Hr	0.01029 **	0.00314	0.28322	0.08627	0.00411 – 0.01648	0.11295 – 0.45348	3.28296	174.00000
sCOD_Removal	23.15617 ***	6.21759	0.28074	0.07538	10.88455 – 35.42778	0.13196 – 0.42952	3.72430	174.00000
pCOD_Washout	0.00086 ***	0.00024	0.29020	0.07951	0.00040 – 0.00133	0.13327 – 0.44713	3.64974	174.00000
Observations	180							
R^2 / R^2 adjusted	0.183 / 0.159							

* $p<0.05$ ** $p<0.01$ *** $p<0.001$

	**M6-NRI**							
Predictors	*Estimates*	*std. Error*	*std. Beta*	*standardized std. Error*	*CI*	*standardized CI*	*Statistic*	*df*
(Intercept)	-26.37880 ***	6.65075	-0.00000	0.06831	-39.50585 – -13.25175	-0.13484 – 0.13484	-0.00000	173.00000
Status_XS	-0.51587 **	0.17420	-0.22224	0.07504	-0.85970 – -0.17205	-0.37036 – -0.07412	-2.96148	173.00000
Status_S	-0.18699	0.17420	-0.08056	0.07504	-0.53081 – 0.15683	-0.22868 – 0.06756	-1.07346	173.00000
Status_XL	0.25543	0.17420	0.11004	0.07504	-0.08839 – 0.59925	-0.03808 – 0.25816	1.46635	173.00000
ml_Methane_per_Hr	0.01029 **	0.00313	0.28322	0.08623	0.00411 – 0.01648	0.11302 – 0.45342	3.28439	173.00000
sCOD_Removal	23.15617 ***	6.21488	0.28074	0.07535	10.88942 – 35.42291	0.13202 – 0.42946	3.72593	173.00000
pCOD_Washout	0.00086 ***	0.00024	0.29020	0.07948	0.00040 – 0.00133	0.13333 – 0.44707	3.65134	173.00000
Observations	180							
R^2 / R^2 adjusted	0.188 / 0.160							

* $p<0.05$ ** $p<0.01$ *** $p<0.001$

	M7-NRI							
Predictors	*Estimates*	*std. Error*	*std. Beta*	*standardized std. Error*	*CI*	*standardized CI*	*Statistic*	*df*
(Intercept)	-26.12337 ***	6.66486	-0.00000	0.06845	-39.27882 – -12.96792	-0.13511 – 0.13511	-0.00000	172.00000
Status_XS	-0.77131 ***	0.20155	-0.33228	0.08683	-1.16913 – -0.37348	-0.50366 – -0.16090	-3.82693	172.00000
Status_S	-0.44242 *	0.20155	-0.19060	0.08683	-0.84025 – -0.04460	-0.36198 – -0.01921	-2.19513	172.00000
Status_M	-0.19955	0.20155	-0.08597	0.08683	-0.59738 – 0.19827	-0.25735 – 0.08541	-0.99012	172.00000
Status_L	-0.31131	0.20155	-0.13411	0.08683	-0.70913 – 0.08652	-0.30549 – 0.03727	-1.54458	172.00000
ml_Methane_per_Hr	0.01029 **	0.00314	0.28322	0.08640	0.00410 – 0.01649	0.11267 – 0.45376	3.27781	172.00000
sCOD_Removal	23.15617 ***	6.22735	0.28074	0.07550	10.86429 – 35.44804	0.13172 – 0.42977	3.71846	172.00000
pCOD_Washout	0.00086 ***	0.00024	0.29020	0.07964	0.00040 – 0.00133	0.13301 – 0.44739	3.64402	172.00000
Observations	180							
R^2 / R^2 adjusted	0.190 / 0.157							

* *p<0.05   ** p<0.01   *** p<0.001*

Subset Regression Analysis – Nearest Taxa Index (NTI)

Model Number	Model	Cross-validation Errors
M5	NTI ~ Status_XS + Status_S + ml_Methane_per_Hr + sCOD_Removal + pCOD_Washout	0.85443
M4	NTI ~ Status_XS + Status_S + sCOD_Removal + pCOD_Washout	0.85525
M6	NTI ~ Status_M + Status_L + Status_XL + ml_Methane_per_Hr + sCOD_Removal + pCOD_Washout	0.85600
M3	NTI ~ Status_XS + Status_S + sCOD_Removal	0.85640
M7	NTI ~ Status_XS + Status_S + Status_M + Status_L + ml_Methane_per_Hr + sCOD_Removal + pCOD_Washout	0.85984
M2	NTI ~ Status_XS + Status_S	0.86946
M1	NTI ~ Status_M	0.88220

	M1-NTI							
Predictors	*Estimates*	*std. Error*	*std. Beta*	*standardized std. Error*	*CI*	*standardized CI*	*Statistic*	*df*
(Intercept)	4.66592 ***	0.07414	-0.00000	0.07356	4.51961 – 4.81224	-0.14516 – 0.14516	-0.00000	178.00000
Status_M	0.39851 *	0.16579	0.17731	0.07377	0.07135 – 0.72568	0.03174 – 0.32288	2.40373	178.00000
Observations	180							
R^2 / R^2 adjusted	0.031 / 0.026							

* *p*<0.05   ** *p*<0.01   *** *p*<0.001

	M2-NTI							
Predictors	*Estimates*	*std. Error*	*std. Beta*	*standardized std. Error*	*CI*	*standardized CI*	*Statistic*	*df*
(Intercept)	4.93991 ***	0.08412	-0.00000	0.07228	4.77390 – 5.10591	-0.14264 – 0.14264	-0.00000	177.00000
Status_XS	-0.46970 **	0.16824	-0.20899	0.07486	-0.80171 – -0.13768	-0.35671 – -0.06126	-2.79183	177.00000
Status_S	-0.50171 **	0.16824	-0.22323	0.07486	-0.83372 – -0.16969	-0.37095 – -0.07550	-2.98210	177.00000
Observations	180							
R^2 / R^2 adjusted	0.070 / 0.060							

* *p*<0.05 ** *p*<0.01 *** *p*<0.001

	**M3-NTI**							
Predictors	*Estimates*	*std. Error*	*std. Beta*	*standardized std. Error*	*CI*	*standardized CI*	*Statistic*	*df*
(Intercept)	-7.91788	5.58793	-0.00000	0.07142	-18.94586 – 3.11009	-0.14094 – 0.14094	-0.00000	176.00000
Status_XS	-0.46970 **	0.16623	-0.20899	0.07396	-0.79777 – -0.14163	-0.35496 – -0.06302	-2.82551	176.00000
Status_S	-0.50171 **	0.16623	-0.22323	0.07396	-0.82978 – -0.17364	-0.36920 – -0.07726	-3.01807	176.00000
sCOD_Removal	13.16161 *	5.71934	0.16480	0.07162	1.87430 – 24.44891	0.02347 – 0.30614	2.30125	176.00000
Observations	180							
R^2 / R^2 adjusted	0.097 / 0.082							

* *p<0.05* ** *p<0.01* *** *p<0.001*

	**M4-NTI**							
Predictors	*Estimates*	*std. Error*	*std. Beta*	*standardized std. Error*	*CI*	*standardized CI*	*Statistic*	*df*
(Intercept)	-7.73044	5.56854	-0.00000	0.07115	-18.72058 – 3.25970	-0.14042 – 0.14042	-0.00000	175.00000
Status_XS	-0.46970 **	0.16562	-0.20899	0.07369	-0.79656 – -0.14283	-0.35442 – -0.06355	-2.83604	175.00000
Status_S	-0.50171 **	0.16562	-0.22323	0.07369	-0.82857 – -0.17484	-0.36866 – -0.07779	-3.02932	175.00000
sCOD_Removal	12.48867 *	5.71523	0.15638	0.07156	1.20901 – 23.76832	0.01514 – 0.29762	2.18515	175.00000
pCOD_Washout	0.00031	0.00021	0.10888	0.07156	-0.00009 – 0.00072	-0.03236 – 0.25012	1.52145	175.00000
Observations	180							
R^2 / R^2 adjusted	0.109 / 0.089							

* *p<0.05   ** p<0.01   *** p<0.001*

	M5-NTI							
Predictors	*Estimates*	*std. Error*	*std. Beta*	*standardized std. Error*	*CI*	*standardized CI*	*Statistic*	*df*
(Intercept)	-11.92628	6.70155	-0.00000	0.07110	-25.15306 – 1.30051	-0.14032 – 0.14032	-0.00000	174.00000
Status_XS	-0.46970 **	0.16549	-0.20899	0.07363	-0.79633 – -0.14306	-0.35432 – -0.06365	-2.83816	174.00000
Status_S	-0.50171 **	0.16549	-0.22323	0.07363	-0.82834 – -0.17508	-0.36856 – -0.07790	-3.03159	174.00000
ml_Methane_per_Hr	0.00355	0.00316	0.10082	0.08974	-0.00269 – 0.00978	-0.07630 – 0.27795	1.12345	174.00000
sCOD_Removal	15.37595 *	6.26259	0.19253	0.07842	3.01554 – 27.73637	0.03776 – 0.34730	2.45521	174.00000
pCOD_Washout	0.00045	0.00024	0.15558	0.08272	-0.00002 – 0.00092	-0.00767 – 0.31884	1.88095	174.00000
Observations	180							
R^2 / R^2 adjusted	0.116 / 0.090							

** p<0.05   ** p<0.01   *** p<0.001*

	M6-NTI							
Predictors	*Estimates*	*std. Error*	*std. Beta*	*standardized std. Error*	*CI*	*standardized CI*	*Statistic*	*df*
(Intercept)	-12.41198	6.69815	-0.00000	0.07106	-25.63259 – 0.80863	-0.14025 – 0.14025	-0.00000	173.00000
Status_M	0.61023 ***	0.17544	0.27151	0.07806	0.26396 – 0.95650	0.11745 – 0.42558	3.47836	173.00000
Status_L	0.39455 *	0.17544	0.17555	0.07806	0.04827 – 0.74082	0.02148 – 0.32962	2.24894	173.00000
Status_XL	0.45233 *	0.17544	0.20126	0.07806	0.10606 – 0.79860	0.04719 – 0.35533	2.57833	173.00000
ml_Methane_per_Hr	0.00355	0.00316	0.10082	0.08969	-0.00268 – 0.00978	-0.07621 – 0.27786	1.12407	173.00000
sCOD_Removal	15.37595 *	6.25917	0.19253	0.07837	3.02178 – 27.73012	0.03784 – 0.34723	2.45655	173.00000
pCOD_Washout	0.00045	0.00024	0.15558	0.08267	-0.00002 – 0.00092	-0.00759 – 0.31876	1.88197	173.00000
Observations	180							
R^2 / R^2 adjusted	0.122 / 0.091							

* $p<0.05$ ** $p<0.01$ *** $p<0.001$

	M7-NTI							
Predictors	*Estimates*	*std. Error*	*std. Beta*	*standardized std. Error*	*CI*	*standardized CI*	*Statistic*	*df*
(Intercept)	-11.95965	6.71787	-0.00000	0.07126	-25.21973 – 1.30044	-0.14066 – 0.14066	-0.00000	172.00000
Status_XS	-0.43633 *	0.20315	-0.19414	0.09039	-0.83732 – -0.03534	-0.37255 – -0.01572	-2.14781	172.00000
Status_S	-0.46834 *	0.20315	-0.20838	0.09039	-0.86933 – -0.06735	-0.38679 – -0.02997	-2.30538	172.00000
Status_M	0.15790	0.20315	0.07025	0.09039	-0.24309 – 0.55889	-0.10816 – 0.24867	0.77725	172.00000
Status_L	-0.05779	0.20315	-0.02571	0.09039	-0.45877 – 0.34320	-0.20413 – 0.15270	-0.28445	172.00000
ml_Methane_per_Hr	0.00355	0.00317	0.10082	0.08995	-0.00270 – 0.00980	-0.07672 – 0.27837	1.12089	172.00000
sCOD_Removal	15.37595 *	6.27689	0.19253	0.07860	2.98631 – 27.76560	0.03739 – 0.34767	2.44962	172.00000
pCOD_Washout	0.00045	0.00024	0.15558	0.08290	-0.00002 – 0.00092	-0.00806 – 0.31923	1.87666	172.00000
Observations	180							
R^2 / R^2 adjusted	0.122 / 0.086							

* $p<0.05$ ** $p<0.01$ *** $p<0.001$

References

Dray, S., Blanchet, G., Borcard, D., Guenard, G., Jombart, T., Larocque, G., Legendre, P., Madi, N., Wagner, H.H., 2016. adespatial: Multivariate multiscale spatial analysis. R Packag. version 0.0 3.

Foster, Z.S.L., Sharpton, T.J., Grünwald, N.J., 2017. Metacoder: An R package for visualization and manipulation of community taxonomic diversity data. PLOS Comput. Biol. 13, e1005404.

Kassambara, A., 2018. Machine Learning Essentials: Practical Guide in R. CreateSpace Independent Publishing Platform.

Kembel, S.W., Cowan, P.D., Helmus, M.R., Cornwell, W.K., Morlon, H., Ackerly, D.D., Blomberg, S.P., Webb, C.O., 2010. Picante: R tools for integrating phylogenies and ecology. Bioinformatics 26, 1463–1464.

Legendre, P., De Cáceres, M., 2013. Beta diversity as the variance of community data: dissimilarity coefficients and partitioning. Ecol. Lett. 16, 951–963. https://doi.org/10.1111/ele.12141

Lüdecke, D., 2019. sjPlot: Data visualization for statistics in social science. CRAN. https://doi.org/10. 5281/zenodo.1308157

Lumley, T., Miller, A., 2009. Leaps: regression subset selection. R package version 2.9. CRAN.

Max Kuhn, 2005. Building Predictive Models in R Using the caret Package. J. Stat. Softw. 14, 981029. https://doi.org/10.18637/jss.v081.b02

Oksanen, J., Blanchet, F., Kindt, R., Legendre, P., Minchin, P.R., O'hara, R., Simpson, G.L., Solymos, P., Stevens, H.H., Wagner, H., 2015. Vegan: community ecology package. R Package version 2.2-1.

Rohart, F., Gautier, B., Singh, A., Lê Cao, K.-A., 2017. mixOmics: An R package for ‘omics feature selection and multiple data integration. PLOS Comput. Biol. 13, e1005752.

Trego, A.C., Galvin, E., Sweeney, C., Dunning, S., Murphy, C., Mills, S., Nzeteu, C., Quince, C., Connelly, S., Ijaz, U.Z., Collins, G., 2020. Growth and Break-Up of Methanogenic Granules Suggests Mechanisms for Biofilm and Community Development. Front. Microbiol. 11, 1126. https://doi.org/10.3389/fmicb.2020.01126
